# Deciphering the Potential Pharmaceutical Mechanism of Chinese Traditional Medicine (Gui-Zhi-Shao-Yao-Zhi-Mu) on Rheumatoid Arthritis

**DOI:** 10.1038/srep22602

**Published:** 2016-03-03

**Authors:** Lin Huang, Qi Lv, Duoli Xie, Tieliu Shi, Chengping Wen

**Affiliations:** 1 TCM Clinical Basis Institute, Zhejiang Chinese Medicine University, 548 Binwen Road, Hangzhou, Zhejiang, 310000, China; 2 Center for Bioinformatics and Computational Biology, and the Institute of Biomedical Sciences, School of Life Science, East China Normal University, 500 Dongchuan Road, Shanghai, 200241, China; 3 School of Finance and Statistics, East China Normal University, 500 Dongchuan Road, Shanghai, 200241, China; 4 Biological Targeting Diagnosis and Therapy Research Center, Guangxi Medical University, Nanning, Guangxi, 530021, China

## Abstract

Gui-Zhi-Shao-Yao-Zhi-Mu (GSZ) decoction is a Traditional Chinese Medicine (TCM) formula commonly used for the treatment of Rheumatoid Arthritis (RA). The therapeutic effect of GSZ for RA treatment is supported by our clinical retrospective study. To uncover the potential mechanism underlying GSZ formula, we identified 1,327 targets of 673 compounds from 9 herbs that involve in Fc epsilon RI signaling pathway and regulation of immunoglobulin production. Comparison between formula targets with 79 RA drug targets and 675 RA disease genes showed that formula targets covered 31.6% RA drug targets and 19.9% RA disease genes. Formula specific targets presented expression patterns highly similar to the disease genes and drug targets based on the expression profiles of RA samples. Investigation of 10 inferred gene clusters from expression profiles with a target association network revealed that formula specific targets directly or indirectly interacted with disease genes that were essential for immune related biological processes (e.g. inflammatory responses, treatment response of rheumatoid arthritis, etc.). Our result indicated that GSZ disrupted the RA disease dysfunction modules and restored homeostasis in the human body. The systemic approach to infer therapeutic mechanisms of GSZ for RA treatment provides a new insight in the understanding of this TCM formula.

Rheumatoid arthritis (RA) is a systemic autoimmune disease that clinically manifests articular and extra articular dysfunctions such as inflammatory response of synovium, turgescence of synovial cells, bone erosions, and cartilage destruction, etc^1^. The intricate etiopathogenesis of RA is related to T cell, B cell, TLRs (Toll-like receptors) and virus^2^,^3^,^4^. The existing treatment of this disease includes non-steroidal anti-inflammatory drugs (NSAIDs), disease modifying anti-rheumatic drugs (DMARDs), glucocorticoids and biologic agents to attenuate symptom and prevent the joint deformation^5^. However these drugs have long-term side effects such as increased risk of infections, potential fatal liver damage among others^6^. Specifically, a commonly used DMARD, methotrexate is associated with a number of adverse effects^7^; another DMARD for RA^8^, leflunomide (LEF), also has unavoidable side effects^9^. In addition, genetic and physiological differences of patients also limit the therapeutic effects of these drugs in RA treatment.

Compared with the above conventional treatment, Traditional Chinese Medicine (TCM), especially herbal medicine, provides a more flexible approach for individualized medicine. In 2015 the discovery of TCM herb, artemisinin, gathered international attention for the treatment of malaria^10^. However, herbal medicine has been in use for more than 3000 years. The various combinations, dosages and compatibility of herbs are modified according to the specific physiological conditions of diseases and patients. Therefore, herbal medicine significantly improves individualized treatment by utilizing the additive or synergistic activities of independent herb, and balancing or neutralizing the toxic effects of herbal components in the mixture.

Among these effective formulas, Gui-Zhi-Shao-Yao-Zhi-Mu (GSZ) decoction, an important formula developed by Zhongjing Zhang, has been used for RA treatment in China since Han Dynasty^11^. The recommended herbal combination of GSZ is *Cinnamomum cassia* (*Gui Zhi*, 12 g), *Paeonia albiflora* (*Shao Yao*, 9 g), *Ephedra sinica* (*Ma Huang*, 12 g), *Anemarrhena asphodeloides* (*Zhi Mu*, 12 g), *Radix Glycyrrhizae Preparata* (*Zhi Gan Cao*, 6 g), *Saposhnikovia divaricata* (*Fang Feng*, 12 g), *Aconitum carmichaeli* (*Fu Zi*, 10 g), *Zingiber officinale* (*Sheng Jiang*, 15 g) and *Atractylodes macrocephala* (*Bai Zhu*, 15 g). The main pharmacological effects of these nine herbs are anti-inflammatory and analgesia. For examples, *Anemarrhena asphodeloides* and *Cinnamomum cassia* affect NF-κB (nuclear factor of kappa light polypeptide gene enhancer in B-cells) pathway^12^,^13^; *Paeonia albiflora* has hepatoprotective and immuno-regulatory activities^14^; *Ephedra sinica* and *Aconitum carmichaeli* alleviate pain^15^. Although anti-inflammatory functions of singular herb has been explored extensively, precise mechanism of TCM herbal combination in formula remain elusive because of the multi-target characteristics.

Systematic approaches provide novel insights to medical studies, especially for herbal medicine^16^. Protein-protein interactions (PPIs), one of the approaches, plays a vital role in bioprocess and it is commonly applied in bioinformatics and medical studies. For example, it was used to define the interactions between proteins in outer membrane^17^. Moreover, the interaction of proteins can help in understanding the cells regulatory among the genome-wide^18^. Academic resources, such as Traditional Chinese Medicine Integrated Database (TCMID, http://www.megabionet.org/tcmid/)^19^, OMIM (http://www.omim.org)^20^ and other databases enable systematic researches to help generate novel insights to medical studies, especially for TCM formulas. For example, by combining microarray gene expression profiling with Connectivity Map, Si-Wu-Tang was identified as an Nrf2 (nuclear factor-erythroid 2-related factor) activator and phytoestrogen^21^; a multi-level integration of herbs, compounds and targets uncovered the pharmacological mechanism of Sheng-ma-bie-jia-tang formula for Systemic Lupus Erythematosus treatment^22^. Some recent researches focused on the comparison between disease genes and relevant drugs to understand the pharmacological mechanism of Chinese herbal medicine^4^,^23^. Information from systematically integrative analysis of different types of data, such as PPIs, provides us a deeper insight into the relationship between diseases and into the understanding of human diseases^24^,^25^.

To explore the pharmaceutical potential mechanism of GSZ decoction for RA treatment, we systematically investigated all the known compounds and targets found in this formula. Comprehensive comparisons among GSZ targets, RA drug targets and RA disease genes further validated the effectiveness of GSZ formula. This systematic approach to investigate the molecular mechanism improves the understanding of the processes and efficacy of this ancient formula.

## Results

### The herbs, compounds and targets of the GSZ formula

**Figure 1:**
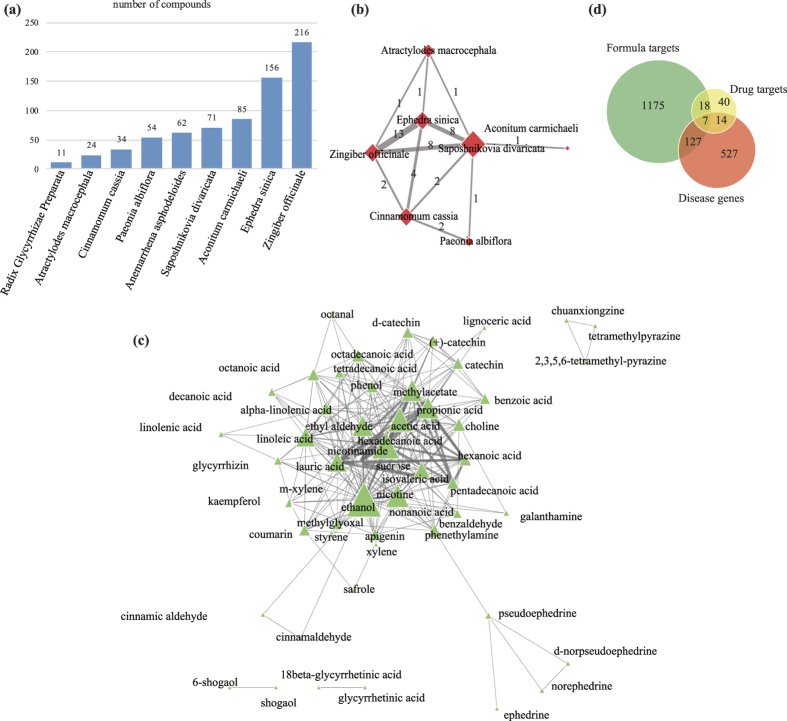
The relationships between herbs, compounds and targets. (**a**) The number of compounds in each herbs. The numbers of compounds for herbs were 11 to 216. (**b**) The relationships between the 7 herbs sharing the same compounds. The links between herbs were constructed with the same compounds in Supplementary Table S1. The width of the edge is related to the similarity between herbs in compounds. (**c**) The relationship between compounds. The network shows the relationship between compounds sharing more than 5 targets. The links between compounds were constructed with the same targets. The width of the edge is related to the similarity between targets in compounds. (**d**) Distribution of targets in GSZ decoction, drug targets and disease genes.

In TCM theory, the main functions of herbs in one formula can be classified as Jun (Monarch), Chen (Minister), Zuo (Assistant) and Shi (Guide) with the importance in descending order^26^. For GSZ formula, *Cinnamomum cassia, Paeonia albiflora, Anemarrhena asphodeloides* and *Aconitum carmichaeli* play dominant roles in the treatment, while the other herbs are auxiliary^18^. We first made descriptive study of the data about herbs, compounds and targets to have a general idea of these important formula components, which is the foundation of all the following analyses. Within the nine herbs, a total of 673 different compounds were retrieved from TCMID (Supplement Table S1, Fig. 1a), and functions of these unique compound components were explored to illustrate the efficacy of the herbs. Aconitine and its derivatives (typical compounds of *Aconitum carmichaeli*) are antinociceptive in different assays without any habit-forming potential^27^. Cinnamaldehyde (the major active ingredient in *Cinnamomum cassia*) participates in anti-inflammation by regulating inflammatory and anti-inflammatory mediators^28^. *Paeonia albiflora* is a well-known herb for treating RA mainly due to the function of its compound TGP (total glycosides of paeony) that are capable of hepatoprotective and immuno-regulatory activities^29^. *Anemarrhena asphodeloides* contains unique compounds, timosaponin a3 and sarsasapogenin, that are related to anti-inflammation processes^30^. In addition, unique compounds in assistant and guide herbs also play a specific roles in the formula’s efficacy. For example, 10-gingerol, 8-shogaol and 10-shogaol extracted from *Zingiber officinale* strongly inhibit COX-2 (cyclooxygenase 2) and thereby reduce inflammation^31^. Those herbs with less or no compounds in common could play distinct roles in formula, e.g. *Aconitum carmichaeli* and *Saposhnikovia divaricata*.

Among 673 compounds, 644 of them are unique compounds that belong to individual herb; the remaining 29 compounds are common between different herbs, suggesting their functional coordination in related biological processes. Both *Radix Glycyrrhizae Preparata* and *Anemarrhena asphodeloides* only contain unique compounds while the compounds in common between the rest seven herbs lead to 11 herb association pairs (Fig. 1b). Among the seven herbs, *Ephedra sinica* and *Zingiber officinale* share 13 compounds, including beta-eudesmol, citronellol, camphor, etc., which are related to antimicrobial properties^32^,^33^,^34^. *Ephedra sinica* and *Saposhnikovia divaricata* contain eight same compounds that cover antibiosis, antiviral, anti-inflammatory and antiangiogenic activity^35^,^36^,^37^,^38^, e.g. beta-eudesmol, beta-pinene, hexanal, etc.

The common targets between different compounds indicate potential synergistic effects. Due to lacking of effective methods to study multi-compounds herbs, the targets of many compounds remain unknown^39^. Thus we focus on the known compounds and targets in this formula for the subsequent analyses. Among the 673 compounds, 147 of them have 1,327 targets. 662 targets were shared by 114 compounds, which connect different compounds to form an compound association network (Fig. 1c). The compound association network was centered by the closely associations between 7 compounds (Supplement Figure S1). Two of them (propionic acid and lauric acid) belong to nonselective NSAIDs for RA^40^ and can adjust RA disease activity independently^41^. Some effective compounds separated from the centered cluster, e.g. tetramethylpyrazine and norephedrine, which ameliorate the inflammation of arthritis.

The 662 shared targets and 665 unique targets of the compounds likely contribute to synergistic and distinct effect respectively. For instance, a shared targets, TNF (tumor necrosis factor), which is commonly used in clinical treatment of RA^42^, is activated by 35 different compounds of GSZ. In contrast, 665 targets are unique to individual compound or herb, indicating the distinct effect of the compound or herb. For example, HSD11B2 [hydroxysteroid (11-beta) dehydrogenase 2] is the only target of three compounds (glycyrrhizin, 18 beta-glycyrrhetinic acid and glycyrrhetinic acid) of *Radix Glycyrrhizae Preparata* and is involved in the activation of synthetic glucocorticoids that is also one of the main drugs to treat RA^43^. Since GSZ formula takes effects through interacting with related targets with specific function, we systematically investigated their targets to uncover their roles in RA treatment.

### Functional analysis of the GSZ targets

We undertook the GO and pathway enrichment analyses of 1,327 targets to explore their general functions. The targets were significantly enriched in 37 GO terms of immune response and cell differentiation (Supplement Table S2), e.g. leukocyte differentiation (p = 2.62E-04), myeloid cell differentiation (p = 2.75E-04), positive regulation of lymphocyte activation (p = 4.07E-04), etc. These terms can be classified into 9 highly connected groups (Supplement Figure S2), e.g. positive regulation of lymphocyte activation, regulation of production of molecular mediator of immune response, regulation of immunoglobulin production, etc. All of these enriched GO terms and groups suggest the essential function of the targets in immunologic process.

These targets were also enriched in 58 pathways (Supplement Table S3). Identification of these pathways that are closely correlated to RA improves our understanding of the functional contributions of the targets to RA treatment. For example, CLA (Conjugated linoleic acids, Linoleic acid metabolism pathway [p = 9.20E-19]) affects a range of inflammatory conditions and helps to alleviate the symptoms of RA^15^. Glycerophospholipid metabolism (p = 6.80E-08), fatty acid metabolism (p = 4.20E-09), tryptophan metabolism (p = 8.90E-05) and linoleic acid metabolism are all involved in the process of RA^5^. An immune system pathway, Fc epsilon RI signaling pathway (p = 7.90E-06), interacts with IgE (immunoglobulin E) to regulate immune processes^44^. Three endocrine system pathways (Adipocytokine signaling pathway [p = 8.30E-11], PPAR signaling pathway [p = 6.30E-16] and GnRH signaling pathway [p= 8.60E-08]) are involved in the inflammatory joint disease therapy^45^. The pathway enrichment analyses for the targets of those herbs suggest that the compounds mainly interact with those targets involved in RA disease.

### Comparison between GSZ targets, other RA drug targets and RA disease related proteins

To further explore the underlying pharmaceutical mechanisms for GSZ formula, we compared its 1,327 targets with the 79 targets of 30 FDA approved RA drugs (Supplement Table S4) and 675 reported RA disease genes (Supplement Table S5) (Fig. 1d). We noticed that 134 and 25 formula targets overlap with RA disease genes and drug targets respectively, while 7 formula targets were both RA disease genes and drug targets (Supplement Table S6).

**Figure 2:**
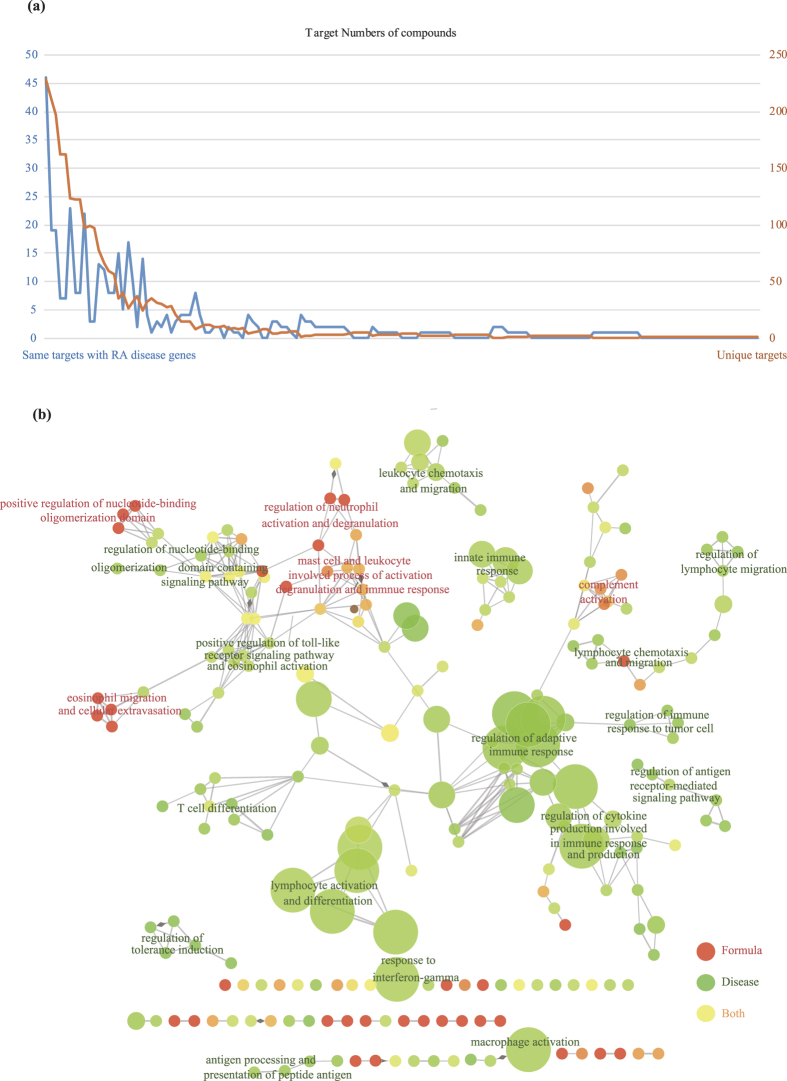
Relationship between formula targets and disease genes. (**a**) The number of targets in different compounds. The orange line stands for the number of unique targets of the compounds while the blue line represents the targets that overlap disease targets. (**b**) The ratio of formula targets and disease genes in immune system processes. The network was generated with ClueGO in Cytoscape.

134 targets of 93 compounds were RA disease genes (Fig. 2a), e.g. TNFSF11 (tumor necrosis factor superfamily, member 11) and IL18 (interleukin 18), etc. To further compare the functional relationships between GSZ formula targets and RA disease genes, we investigated the biological processes of the genes involved (Fig. 2b). Notably, disease genes and formula targets are mainly involved in distinct immune system processes: T cell differentiation, lymphocyte activation and differentiation, regulation of adaptive immune response for disease genes; positive regulation of nucleotide-binding oligomerization domain, eosinophil migration and cellular extravasation and regulation of neutrophil activation and degranulation for formula targets. The shared targets participate in mast cell and leukocyte involved process of activation, degranulation and immune response, positive regulation of toll-like receptor signaling pathway and eosinophil activation, which connected formula specific targets to RA disease genes, indicating the influence of GSZ formula on specific RA disease genes and immune processes.

**Figure 3:**
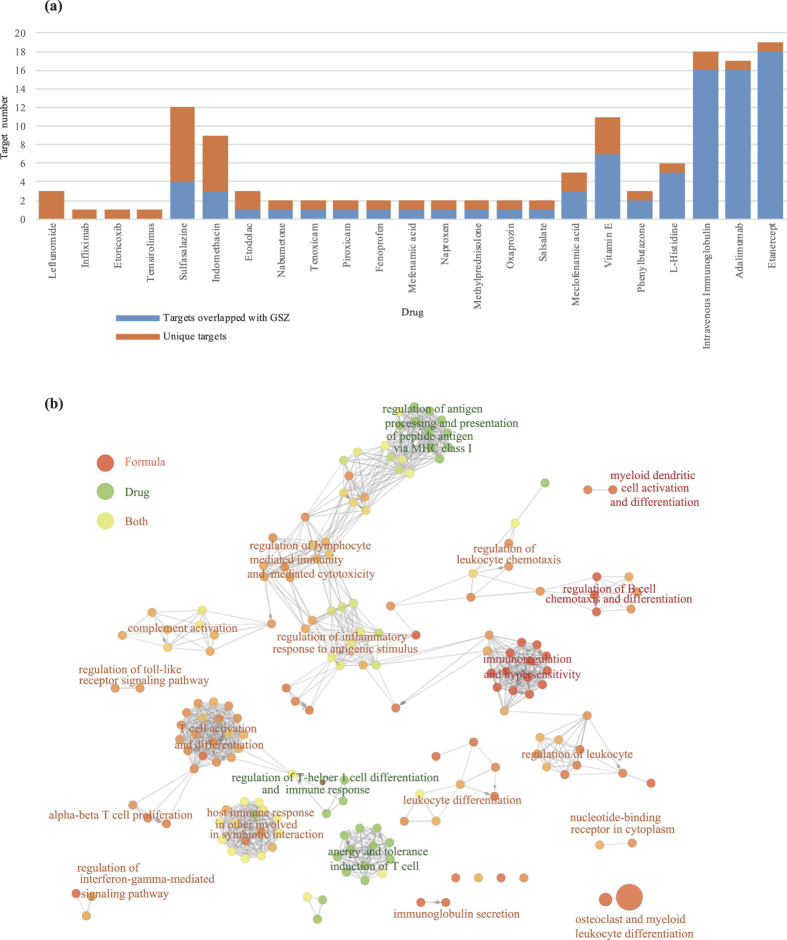
Relationship between formula targets and drug targets. (**a**) The number of targets in different drugs. The orange part represents unique targets of the every drug while the blue one represents the targets that overlap GSZ formula. (**b**) The ratio of formula targets and drug targets in immune system processes. The network was generated with ClueGO in Cytoscape.

For 25 formula targets sharing with the targets of 30 FDA proved RA drugs (Fig. 3a), six of them cover the targets of four current prevalent drugs (LEF, Temsirolimus, Etoricoxib and Infliximab), e.g. TNF, DHODH (dihydroorotate dehydrogenase [quinone]), MTOR (mechanistic target of rapamycin [serine/threonine kinase]), PTGS2 (Prostaglandin G/H synthase 2), AHR (aryl hydrocarbon receptor), PTK2B (protein-tyrosine kinase 2-beta). The functional correlations between formula targets and drug targets (Fig. 3b) suggest that the drug targets mainly participated in regulation of antigen processing and presentation of peptide antigen via MHC class I, anergy and tolerance induction of T cell and regulation of T-helper 1 cell differentiation. With GSZ formula targets, regulation of T-helper 1 cell differentiation and immune response was extended to alpha-beta T cell proliferation process by T cell activation and differentiation processes. Also, regulation of antigen processing and presentation of peptide antigen via MHC class I was expanded to regulate B cell chemotaxis and differentiation by regulation of lymphocyte mediated immunity and mediated cytotoxicity, regulation of inflammatory response to antigenic stimulus, and regulation of leukocyte chemotaxis. These results suggest the complementary function of GSZ formula targets to the existing RA drug targets, which makes the treatment more systematic.

### Validation of GSZ targets in large scale expression profiles of RA patients

**Figure 4:**
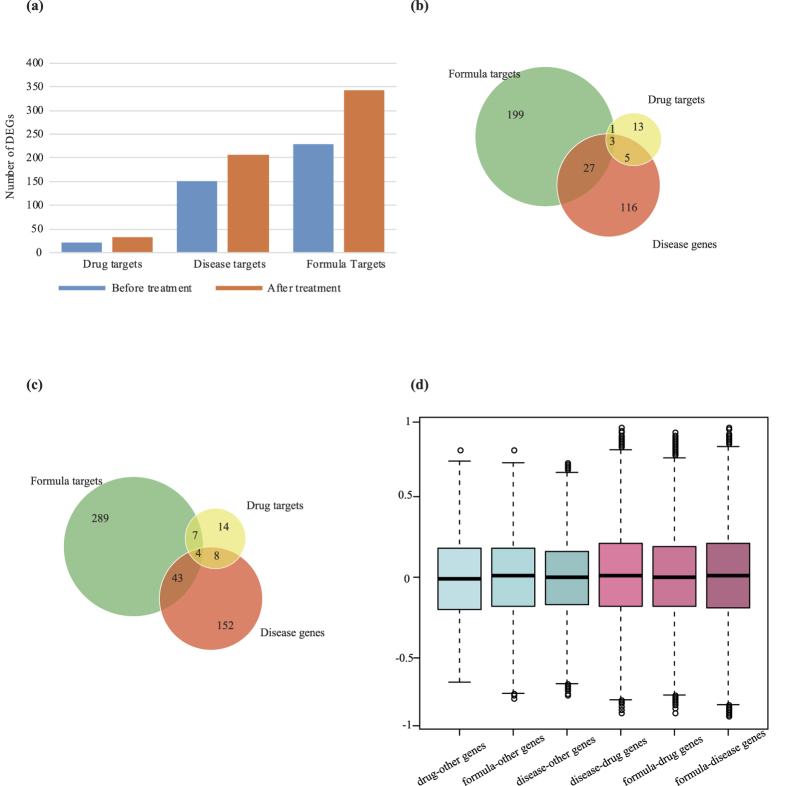
Comparison of quantitative characters of GSZ targets, drug targets and RA disease genes. (**a**) The changes in numbers of differentially expressed gene (DEG) during treatment on RA samples. (**b**) The distribution of GSZ targets, drug targtes and RA genes before treatment. (**c**) The distribution of GSZ targets, drug targtes and RA genes after treatment. (**d**) The correlated coefficients between gene pairs, drug-other genes, formula-other genes, disease-other genes, disease-drug genes, formula-drug genes and formula-disease genes.

To further evaluate and compare quantitative characters of GSZ targets, drug targets and RA disease genes, we investigated these genes using large scale expression profiles related to RA (methods). We discovered that the number of differentially expressed genes (DEGs) in the three datasets, GSZ targets, drug targets and RA disease genes, all increased in treated RA samples (Fig. 4a). The similar expression behaviors of these three gene sets suggest common function between them. Specifically, 343 formula targets were differentially expressed in the samples after drug treatment. The number of differently expressed targets was increased by 1.5 times compared to RA samples before treatment, which is in constant with the changes of the number of differentially expressed disease targets and drug targets (1.4 and 1.5 times respectively). In addition, we found that the most changes of drug targets and disease genes were also included in GSZ formula targets, indicating GSZ formula covers most effective function of other drugs and RA related genes. Before treatment, 18.2% (4/22) and 19.9% (30/151) of DEGs about drug targets and disease genes were the GSZ targets (Fig. 4b), while after treatment, the numbers increased to 33.3% (11/33) and 22.7% (47/207) respectively (Fig. 4c). Using these expression data, we also discovered that in DGEs, the expression of over 98% of GSZ targets, drug targets and RA disease genes were recovered after drug treatment (Supplement Figure S3), indicating the directional function of these targets.

We further confirmed the expression similarity by computing the correlated coefficients between genes pairs (methods, Fig. 4d). As expected, most gene pairs were not correlated in expression, with the distribution of correlated coefficients between −0.2 and 0.2. However, correlated coefficients of the several gene pairs between GSZ targets, drug targets and RA disease genes were higher (>0.75 or <−0.75) compared to the rest genes (<0.75 or >−0.75). The significant correlation of the three gene-sets further indicates that the expression of GSZ targets on RA samples is quite similar to both drug targets and RA disease genes.

### Target association network of RA revealing the mechanism of GSZ treatment

**Figure 5:**
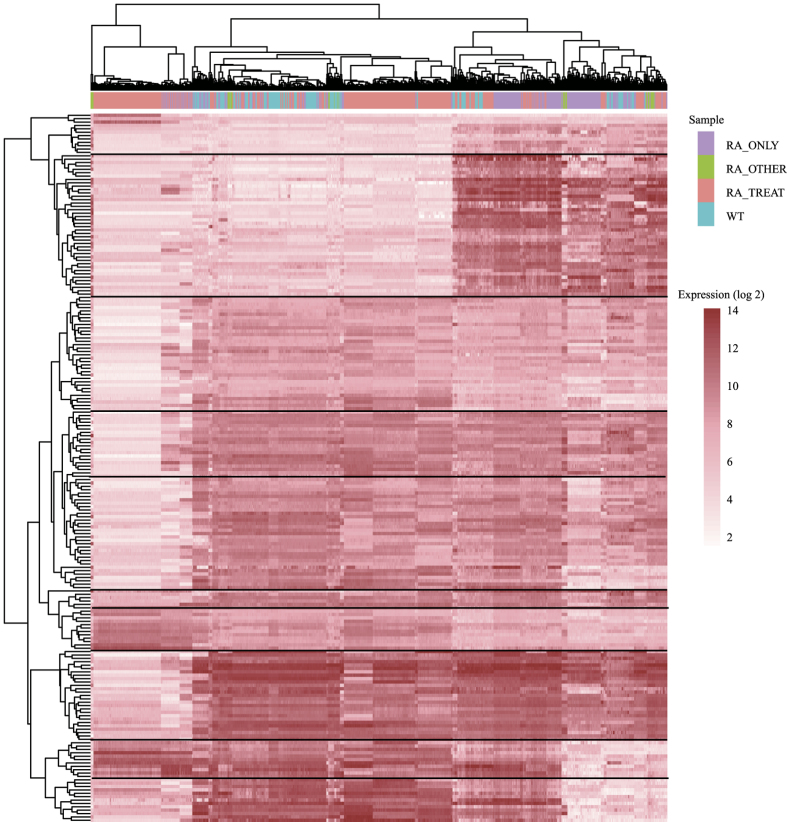
Expression profiles of co-expressed DEGs. The expression of genes in 4 different groups of RA sample, e.g. RA_ONLY, RA_OTHER, RA_TREAT, WT.

**Figure 6:**
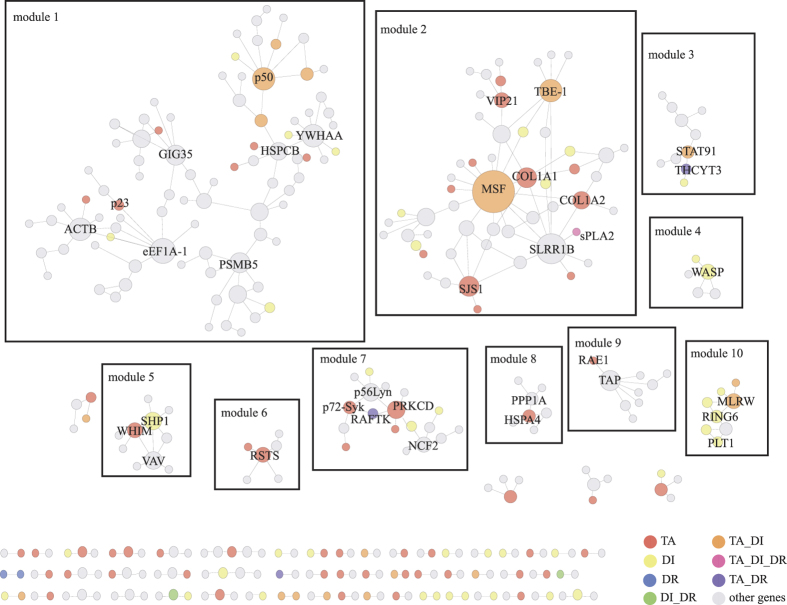
The PPI network of the GSZ formula targets, disease genes, drug targets and other connective targets. The network was generated with ClueGO in Cytoscape. (red: formula targets; yellow: disease genes; blue: drug targets; green: disease genes and drug targets; orange: formula targets and disease genes; pastel pink: formula targets, disease genes and drug targets; gray: other genes).

From the expression profiles of co-expressed DEGs, we discovered 10 gene clusters with similar expression over all samples (Fig. 5). These genes clusters have distinct expression characters, but not specific to the classification of samples, indicating specific function of the gene clusters and individual characters of patients. To further investigate the function of these clusters, we integrated protein-protein interactions (PPIs) with gene co-expression to construct a target association network of RA (Fig. 6).

The modules in the network show that the GSZ targets directly or indirectly interact with RA disease genes, by which GSZ targets could take pharmacological effects in RA treatment. For example, module 1 includes genes that GSZ targets (red) and RA disease genes (yellow) that were connected by other genes (gray). Nuclear factor of kappa light polypeptide gene enhancer in B-cells 1 (p50), defined as both RA disease gene and GSZ target, could be an essential nodes of this cluster by direct interactions with seven genes (Supplement Table S7). The seven genes are involved in cell growth, inflammatory responses, p53-mediated responses, and the apoptosis of rheumatoid synovial fibroblasts^28^,^30^,^46^, that is in accordance with the extensive biological function of p50 in immune homeostasis^25^. In module 10, two formula targets (MLRW [major histocompatibility complex, class II, DR alpha] and HLA-DRB1 [major histocompatibility complex, class II, DR beta 1]) directly or indirectly interact with five disease genes (Supplement Table S8) that all belong to the HLA class II and were related to incidence, mortality, and treatment response of rheumatoid arthritis through biological process about peptides^17^,^47^,^48^.

In addition, we discovered that the targets in GSZ extended the function of specific RA drug targets, e.g. the extension of sPLA2 (phospholipase A2, group IIA, drug target of Indomethacin) to GSZ targets and RA disease genes in module 2. In module 2, fibronectin 1 (FN1, also known as MSF), decorin (DCN, also known as SLRR1B) and matrix metallopeptidase 2 (MMP-2, also known as TBE-1) might be the core members of module 2 by connecting other 50 GSZ targets or RA disease genes in this module. MSF, inhibiting apoptosis and increasing proinflammatory cytokine secretion of fibroblast-like synoviocytes in RA^32^, directly interacted with seven RA disease genes (Supplement Table S9). These seven disease genes participate in inflammatory cell infiltration, inflammasome, destructive synovial tissue, angiogenesis process and pannus formation of RA^34^,^37^,^43^. In module 7, RAFTK (protein tyrosine kinase 2 beta, drug target of LEF) directly interact with two GSZ targets and indirectly associate with more GSZ targets (p72-Syk, spleen tyrosine kinase; nPKC-delta, protein kinase C, delta) and RA disease genes. Interactions in module 7 expands the pharmacological effects of RAFTK to more systematic way by the associated genes, e.g. cell fate, immune response of monocyte subsets, inflammatory milieu in bone, integrin and chemokine signaling^49^,^50^.

### Clinical performance of GSZ in RA patient treatment

We also used clinical data to further validate the efficacy of GSZ formula. The observational study aimed to focus on the effectiveness of GSZ formula with recommended herbal combination for RA treatment. We reviewed the medical records of 10 patients who suffered from finger joint destruction and strictly followed the treatment procedure for more than 6 months. Clinical indexes were collected from three time points at week 6, 8 and 14; the indexes, included rheumatoid factor (RF), erythrocyte sedimentation rate (ESR) and c-reactive protein (CRP) (Supplement Table S10). During the process, no liver damage case was observed. The serum level of improvement in rheumatoid factor showed significant variation during intervention of GSZ formula with p <0.01, but there was no statistical difference in the levels of ESR (p  = 0.32) and CRP (p = 0.8). The results suggest that GSZ formula is an effective therapy, and can enhance the pharmacological effectiveness by decreasing the level of RF and thus effectively protect cartilage as well as control finger joint destruction.

## Discussion

In this study, we validated the efficacy of GSZ formula with comparisons of RA drug targets and RA disease genes, and further explored the potential molecular mechanism of GSZ decoction through a systematic approach.

ESR and CRP are often tested together to aid the diagnosis of inflammatory disease or serious infection^53^. RF is one of the diagnostic items in 2010 American College of Rheumatology/European League Against Rheumatism classification criteria and it is both sensitive and specific in RA patients^52^. RF positivity and levels are closely related to the severity of finger destruction^53^ while extra-articular manifestations and a strong acute phase are indicated by levels of ESR and CRP^54^. The result of clinical performance of GSZ in RA patient treatment suggested that GSZ may be a approach to alleviate the joint destruction of RA patients but not to the extra-articular manifestations.

The function of nine herbs was analyzed with detailed herb related compounds and compound related targets to investigate the underlying mechanism of GSZ formula. We discovered that the shared compounds are of great importance in inflammation or RA treatment. For example, β-eudesmol (a compound in *Atractylodes macrocephala, Saposhnikovia divaricata, Ephedra sinica* and *Zingiber officinale*) was a potential therapy in mast cell-mediated inflammation and an inhibitor of Cytochrome P450 2C9 which accepted responsibility for the metabolism of NSAID^55^; Camphor (a compound in *Cinnamomum cassia, Ephedra sinica* and *Zingiber officinale*) has been used in clinical trial for RA treatment for a long time^56^; β-sitosterol can effect as an anti-inflammation through significantly suppressing the T-cell immune response^57^. Specific compounds in each herb are also closely related to RA. Using *Radix Glycyrrhizae Preparata* as an example, which is found to contain 11 compounds that act on lung tissue, therefore suggesting it as an effective herb to treat RA patients with interstitial lung disease^58^.

Function analysis of GSZ targets suggests that the therapeutic effect of GSZ decoction may result from the regulation of Fc epsilon RI signaling pathway, adipocytokine signaling pathway, and lipid metabolism that are related to mediator release, inflammatory pathologies, antigen-driven autoimmune responses, regulation of whole-body lipid metabolism and matrix degradation in the human joint^59^,^60^,^61^. We also discovered that GSZ targets are overlapped with RA drugs targets and known RA disease genes, which indicates the similar function in immunity adjustment and anti-inflammation, e.g. T cell activity that leads to the development of autoimmune such as RA^62^, MHC class I chain-related gene A (MICA) which have an effect on RA severity^63^.

Until now medicine that were characterized as a single target or a few targets, such as MTX, cannot satisfy the treatment of rheumatoid disease with complicated pathogenesis. In clinical practice, patients are conventionally treated by combining various categories of drugs that have systematically pharmacological effects. However, formulas like GSZ decoction can simultaneously target hundreds of disease proteins, which therefore is more likely to satisfy the therapeutic outcomes for rheumatoid disease. Western medicine is only effective for partial patient population, and TCM therapy has better performance on individualized therapy. Network-based module analyses efficiently illustrate the underlying mechanism of GSZ formula. Compared with FDA approved drugs, GSZ formula targets more RA disease proteins to form a more complex target interaction network in RA treatment, suggesting the systematically pharmaceutical effect of this TCM formula. This is one of the many advantages of TCM in the treatment of complicated diseases.

The complex pharmacological processes of herbal multi-targets characteristics is challenging to research. The traditional study based on a single compound or a single target has inherent limitation and therefore we turned to systemic bioinformatics method. This method provides a novel way to investigate the therapeutic effects of GSZ decoction in RA treatment and serves as a good example for revealing the mechanisms of TCM formulas on treating other diseases.

## Materials and Methods

### Collection of GSZ data, other RA drugs and known RA diseases proteins

The compounds of nine herbs and their related targets were downloaded from TCMID for further analysis and targets were filtered by recommended confidence range defined by STITCH (low confidence: scores <0.4; medium: 0.4 to 0.7; high: >0.7)^64^. Based on these scores, 1,327 highly confident targets with comprehensive scores >0.7 were maintained. Genes associated with RA were collected from three databases: OMIM (http://www.omim.org)^20^, GAD (http://geneticassociationdb.nih.gov/)^65^ and KEGG (http://www.kegg.jp)^66^. Drug related targets were retrieved from DrugBank 4.0 (http://www.drugbank.ca)^67^. The three groups’ targets were analysis using Cytoscape 3.2.1 with ClueGo plugin^68^.

### GO enrichment analysis

DAVID Bioinformatics Resources 6.7 (http://david.abcc.ncifcrf.gov/)^69^, a comprehensive set of functional annotation tools for understanding the biological meaning behind large lists of genes, was used to carry out pathway enrichment for the 1,327 genes targeted by GSZ decoction. Gene ontology analysis was implemented by Cytoscape 3.2.1 with ClueGo plugin. Enriched GO terms and pathways were defined as those with adjust P <0.05.

### Microarray data processing of RA samples

The expression matrix for each series of 1080 microarrays were collected from GEO database, and then classified as 174 normal samples, 312 RA disease samples, 551 RA treatment samples and 42 other RA related samples (Supplement Table S11). Using the annotations of microarray platforms, expressions of probes were transformed into GeneID. Missing values were excluded in further analyses. Then, expression values were normalized by normalize.quantiles function in R. Before and after treatments comparisons were made between normal-RA disease samples and RA disease-treatment samples respectively. Differentially expressed genes were defined as the ones whose fold change values were larger than 2 and adjust P-value less than 0.05. 364 genes were differentially expressed in RA disease samples compared with normal samples, while 517 genes were differentially expressed in RA treatment samples. Co-expression between gene pairs was measured by Pearson correlation coefficient that was computed by cor function in R. Gene pairs of 10297 genes with Pearson correlation coefficient larger than 0.75 (0.01% of the population) were defined as co-expressed.

### Target association network

The clustering distances of DEGs were computed by Euclidean distances. The graph was generated by pheatmap function in R. Clusters were defined by K-means approach using Kmeans function in R. The overlap of co-expressed gene pairs and protein-protein interactions collected from HPRD (http://www.hprd.org/) and STRING (http://string-db.org/) was combined to construct the RA gene association network. The network was generated by Cytoscape.

### Clinical data collection

This observational study was approved by institutional review boards (IRB) of Zhejiang Chinese Medical University and the methods were carried out in accordance with the approved guidelines. All participants signed an informed consent before they participated in the study. We collected patients’ related information generated between December 2013 and August 2015. The Second affiliated Hospital of Zhejiang University of TCM participated in the investigation. Inspection center of this hospital completed drawing blood of every patient and completed testing the clinical indexes including CRP, ESR and RF. Patient characteristics, stage of disease and other parameters are shown in Supplement Table S10. These patients satisfied 2010 American College of Rheumatology/European League Against Rheumatism classification criteria. At initiation of therapy, age, gender, height, weight and serum levels of RF, ESR and CRP of every patient were recorded. Patients with lack of DMARDs, specifically MTX or LEF, response were chosen and those who suffered either liver or renal damage before TCM treatment were filtered out. Finally, 10 patients met the criteria. The DMARDs administered (and their doses) were recorded at each clinic visit and no other western medicine were used for RA treatment during the whole observation process. The re-tested serum levels of RF, ESR, CRP at 14, 8 and 6 week time points, were compared to the initial data and tested the significance of improvement by paired t.test.

## Additional Information

**How to cite this article**: Huang, L. *et al*. Deciphering the Potential Pharmaceutical Mechanism of Chinese Traditional Medicine (Gui-Zhi-Shao-Yao-Zhi-Mu) on Rheumatoid Arthritis. *Sci. Rep.* **6**, 22602; doi: 10.1038/srep22602 (2016).

## Supplementary Material

Supplementary Information
