# Supplementary material for: Deciphering the Potential Pharmaceutical Mechanism of Chinese Traditional Medicine (Gui-Zhi-Shao-Yao-Zhi-Mu) on Rheumatoid Arthritis
Source: Sci Rep. 2016 Mar 3;6:22602. doi: 10.1038/srep22602 (PMC4776278; doi:10.1038/srep22602)
Supplement: Supplementary Information [file srep22602-s1.pdf]

Deciphering the Potential Pharmaceutical Mechanism of Chinese Traditional Medicine (Gui-Zhi-Shao-Yao-Zhi-Mu) on Rheumatoid Arthritis

Lin Huang[1, #], Qi Lv[2, 3, #], Duoli Xie[1], Tieliu Shi[2, 4*], Chengping Wen[1, *]

[1]TCM Clinical Basis Institute, Zhejiang Chinese Medicine University, 548 Binwen Road, Hangzhou, Zhejiang, 310000, China.

[2]Center for Bioinformatics and Computational Biology, and the Institute of Biomedical Sciences, School of Life Science, East China Normal University, 500 Dongchuan Road, Shanghai, 200241, China.

[3]School of Finance and Statistics, East China Normal University, 500 Dongchuan Road, Shanghai, 200241, China.

[4]Biological Targeting Diagnosis and Therapy Research Center, Guangxi Medical University, Nanning, Guangxi, 530021, China.

[#]These authors contributed equally to this work.

Correspondence and requests for materials should be addressed to T.S. (email: tlshi@bio.ecnu.edu.cn) and C.W. (email: wengcp@163.com).

Supplement Figure S1 The relationship between compounds sharing more than 102 targets.

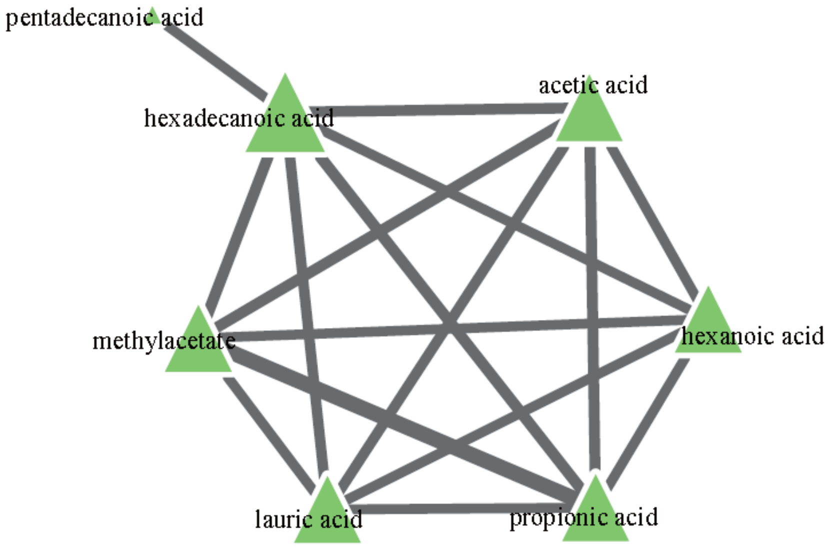

Supplement Figure S2 The group terms of GO enrichment analysis of the formula targets.

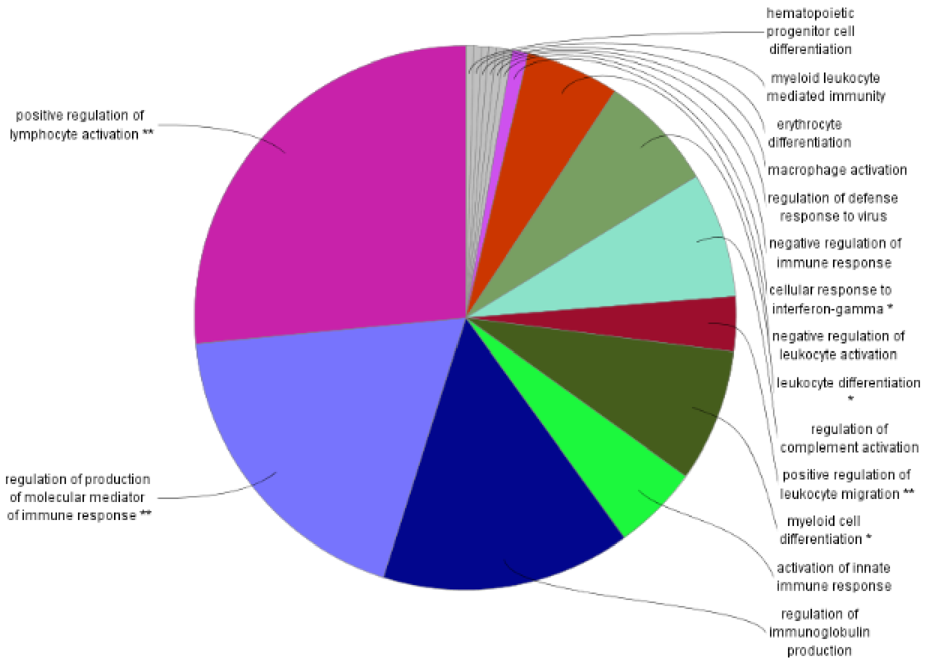

Supplement Figure S3 The expression changes of GSZ targets, drug targets and RA disease genes before and after treatment in RA sample.

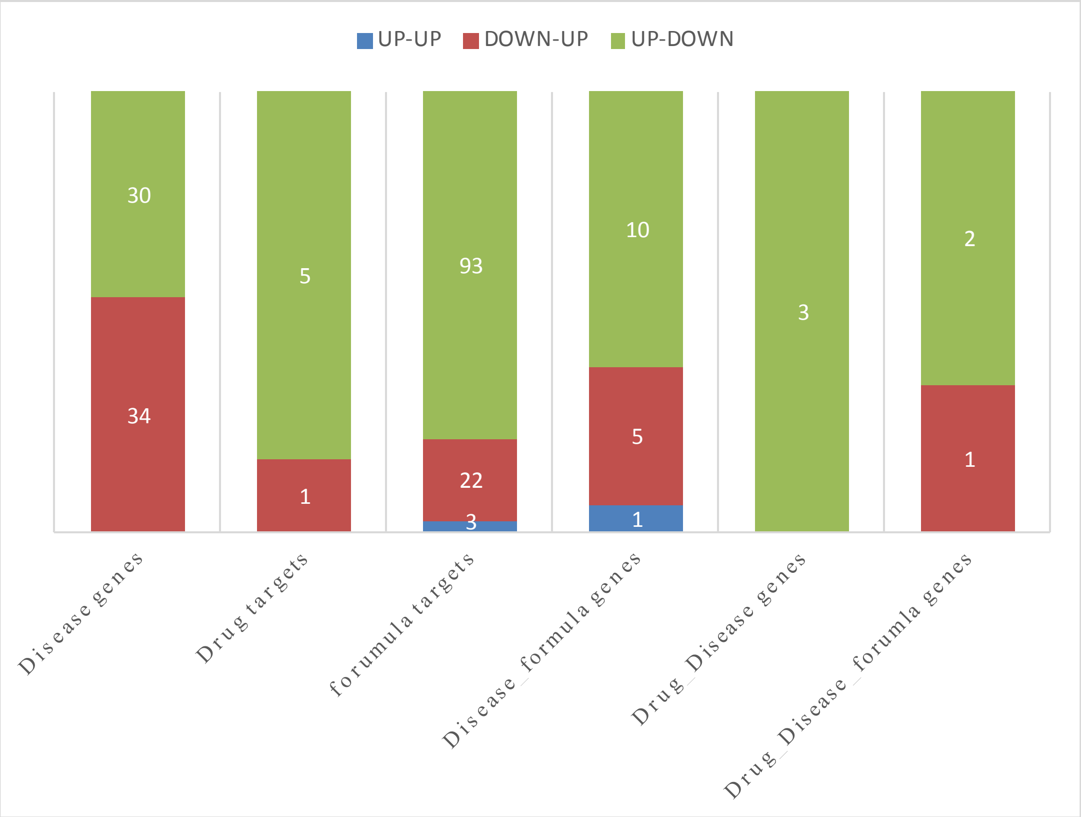

Supplementary Table S1 Detailed description of herbs and compounds in GSZ formula. The compounds of nine herbs were downloaded from TCMID for further analysis. Totally 673 different compounds were retrieved.

**herb**	**compound**
Radix Glycyrrhizae Preparata	rutin
Radix Glycyrrhizae Preparata	schaftoside
Radix Glycyrrhizae Preparata	isoschaftoside
Radix Glycyrrhizae Preparata	18alpha-glycyrrhetinic acid
Radix Glycyrrhizae Preparata	narcissin
Radix Glycyrrhizae Preparata	glycyrrhizin
Radix Glycyrrhizae Preparata	isoquercitrin
Radix Glycyrrhizae Preparata	astragalin
Radix Glycyrrhizae Preparata	ononin
Radix Glycyrrhizae Preparata	18beta-glycyrrhetinic acid
Radix Glycyrrhizae Preparata	glycyrrhetinic acid
Aconitum carmichaeli	6-demethyldesoline
Aconitum carmichaeli	14-o-veratroylneoline
Aconitum carmichaeli	14-o-cinnamoylneoline
Aconitum carmichaeli	delcorine
Aconitum carmichaeli	deltamine
Aconitum carmichaeli	aconitine
Aconitum carmichaeli	aconine
Aconitum carmichaeli	deltaline
Aconitum carmichaeli	bullatine b
Aconitum carmichaeli	bullatine c
Aconitum carmichaeli	lipohypaconitine
Aconitum carmichaeli	lipo-14-o-anisoylbikhaconine
Aconitum carmichaeli	karakoline
Aconitum carmichaeli	higenamine
Aconitum carmichaeli	demethylcoclaurine
Aconitum carmichaeli	neokadsuranic acid b
Aconitum carmichaeli	delphatine
Aconitum carmichaeli	demethyldelavaine a
Aconitum carmichaeli	demethyldelavaine b
Aconitum carmichaeli	neojiangyouaconitine
Aconitum carmichaeli	denudatine
Aconitum carmichaeli	coryneine
Aconitum carmichaeli	8-deoxy-14-dehydro-aconosine
Aconitum carmichaeli	ignavine
Aconitum carmichaeli	lipmesaconitine
Aconitum carmichaeli	3-acetylaconitine
Aconitum carmichaeli	salsolinol
Aconitum carmichaeli	coryneine chloride
Aconitum carmichaeli	2,7-dideacetyl-2,7-dibenzoyl-taxayunnanine f
Aconitum carmichaeli	m-aminophenol
Aconitum carmichaeli	higeamine
Aconitum carmichaeli	delphin
Aconitum carmichaeli	neojianyouaconitine
Aconitum carmichaeli	dictysine

Aconitum carmichaeli	mescaline
Aconitum carmichaeli	13-deoxo-13 alpha-acetyloxy-1-deoxynortaxine i
Aconitum carmichaeli	isotalatizidine
Aconitum carmichaeli	tamarixinol (i)
Aconitum carmichaeli	karanjin
Aconitum carmichaeli	vilmorrianine c
Aconitum carmichaeli	14-o-anisoylneoline
Aconitum carmichaeli	para-aminophenol
Aconitum carmichaeli	karacoline
Aconitum carmichaeli	hypaconitine
Aconitum carmichaeli	mesaconitine
Aconitum carmichaeli	hypaphorine
Aconitum carmichaeli	aconitan
Aconitum carmichaeli	fuzinoside
Aconitum carmichaeli	mesaconine
Aconitum carmichaeli	delbrusine
Aconitum carmichaeli	crassicauline a
Aconitum carmichaeli	delgrandine
Aconitum carmichaeli	deoxyaconitine
Aconitum carmichaeli	delsemine a
Aconitum carmichaeli	delsoline
Aconitum carmichaeli	meta-aminophenol
Aconitum carmichaeli	uracil
Aconitum carmichaeli	songrine
Aconitum carmichaeli	delsemine b
Aconitum carmichaeli	talatisamine
Aconitum carmichaeli	deltoin
Aconitum carmichaeli	dl-demetylcolarine
Aconitum carmichaeli	diacetylacoritine
Aconitum carmichaeli	talatizamine
Aconitum carmichaeli	delavaconitine
Aconitum carmichaeli	benzoylmesaconine
Aconitum carmichaeli	carnosifloside i
Aconitum carmichaeli	carmichaeline
Aconitum carmichaeli	lipodeoxyaconitine
Aconitum carmichaeli	p-aminophenol
Aconitum carmichaeli	o-aminophenol
Aconitum carmichaeli	deoxyandrographolide
Aconitum carmichaeli	benzoylhypaconine
Aconitum carmichaeli	isodelphinine
Aconitum carmichaeli	ortho-aminophenol
Aconitum carmichaeli	delphamine
Aconitum carmichaeli	benzoylaconine
Aconitum carmichaeli	neoline
Aconitum carmichaeli	lipaconitine
Aconitum carmichaeli	delamide
Aconitum carmichaeli	14-deoxy-11,12-didehydroandrographolide

Aconitum carmichaeli	triacetylaconine
Aconitum carmichaeli	hypaconine
Aconitum carmichaeli	delbruline
Aconitum carmichaeli	delbrunine
Ephedra sinica	linolenic acid
Ephedra sinica	camphor
Ephedra sinica	pseudoginsenoside f11
Ephedra sinica	d-pseudoephedrine
Ephedra sinica	4-isopropyl-benzaldehyde
Ephedra sinica	kaempferol-3-rhamnoside
Ephedra sinica	cuminyl alcohol
Ephedra sinica	2-methyl-2-butenal
Ephedra sinica	dibenzofuran
Ephedra sinica	nonadecane
Ephedra sinica	phenanthrene
Ephedra sinica	gamma-terpinene
Ephedra sinica	methyl-7-epiganoderate
Ephedra sinica	alpha-terpineol
Ephedra sinica	isobutyl benzoate
Ephedra sinica	thymol
Ephedra sinica	citronellol
Ephedra sinica	methyl palmitate
Ephedra sinica	heptadecane
Ephedra sinica	tricin
Ephedra sinica	pentadecanoic acid
Ephedra sinica	apigenin-5-rhamnoside
Ephedra sinica	alpha-teripineol
Ephedra sinica	rhatannin
Ephedra sinica	3,4-dimethyl-5-phenyloxazolidine
Ephedra sinica	tetracosane
Ephedra sinica	6-methyl-5-hepten-2-one
Ephedra sinica	kaempferol-7-rhamnoside
Ephedra sinica	octadecanoic acid
Ephedra sinica	hexahydrofarnesylacetone
Ephedra sinica	dimethyl phthalate
Ephedra sinica	acetophenone
Ephedra sinica	leucodelphinidin
Ephedra sinica	2,4-decadienal
Ephedra sinica	methyl acetate
Ephedra sinica	o-methylptelefolonium
Ephedra sinica	d-n-methyl-pseudoephedrine
Ephedra sinica	kaempferol
Ephedra sinica	7-demethylsuberosin
Ephedra sinica	e,e-2,4-decadienal
Ephedra sinica	phenethyl caffeate
Ephedra sinica	(4s,5r)ephedroxane
Ephedra sinica	phytol

Ephedra sinica	(-)-epiafzelechin
Ephedra sinica	norerythrostachaldine
Ephedra sinica	norephedrine
Ephedra sinica	glucoraphanin
Ephedra sinica	p-cymene
Ephedra sinica	nonanoic acid
Ephedra sinica	methyl furoate
Ephedra sinica	myrcene
Ephedra sinica	maokonine
Ephedra sinica	maragenin ii
Ephedra sinica	linalool
Ephedra sinica	o-benzoyl-l- (+) -pseudoephedrine
Ephedra sinica	6-methyl-2-heptanone
Ephedra sinica	hexanal
Ephedra sinica	beta-pinene
Ephedra sinica	pseudoephedroxane
Ephedra sinica	dibutyl phthalate
Ephedra sinica	linoleic acid
Ephedra sinica	1-phenyl-1,2-propanedione
Ephedra sinica	beta-cyclocitral
Ephedra sinica	kaempferol rhamnoside
Ephedra sinica	ephedine
Ephedra sinica	rose oxide
Ephedra sinica	alpha-linolenic acid
Ephedra sinica	methyl benzoate
Ephedra sinica	tetramethylpyrazine
Ephedra sinica	p-isopropyl-benzaldehyde
Ephedra sinica	n-methylephedrine
Ephedra sinica	3-o-β -d-glucopyranosyl-5,9,4'-trihydroxy-8-methoxyflavone
Ephedra sinica	beta-ionone
Ephedra sinica	tetradecanoic acid
Ephedra sinica	guaiazulene
Ephedra sinica	trans-belta-terpineol
Ephedra sinica	trans-2-nonenal
Ephedra sinica	menthyl acetate
Ephedra sinica	di-isobutyl phthalate
Ephedra sinica	n-triacontanol
Ephedra sinica	16-triacontanol
Ephedra sinica	nonanal
Ephedra sinica	2-pentadecanone
Ephedra sinica	dihydro-beta-ionone
Ephedra sinica	naphthalene
Ephedra sinica	decanoic acid
Ephedra sinica	dimethyl naphthalene
Ephedra sinica	1,3,4-trimethyl-3-cyclohexene-1-carboxaldehyde
Ephedra sinica	1,1,3-trimethylcyclopentane
Ephedra sinica	ephedratannina, b

Ephedra sinica	lauric acid
Ephedra sinica	tricosane
Ephedra sinica	2,3,4-trimethyl-5-phenyloxazolidine
Ephedra sinica	alpha-pinene
Ephedra sinica	piperitone
Ephedra sinica	methylphedrine
Ephedra sinica	4,5',8-trimethyl psoralen
Ephedra sinica	chuanxiongzine
Ephedra sinica	styrene
Ephedra sinica	1,5-dimethyl-naphthalene
Ephedra sinica	o-xylene
Ephedra sinica	leucopelargonidin
Ephedra sinica	1,8-cineole
Ephedra sinica	1,4-cineole
Ephedra sinica	safranal
Ephedra sinica	hexadecanoic acid
Ephedra sinica	pseudoephedrine
Ephedra sinica	nor-rubrofusarin
Ephedra sinica	cis-p-2-menthen-1-ol
Ephedra sinica	delta-terpineol
Ephedra sinica	norpseudoephedrine
Ephedra sinica	beta-eudesmol
Ephedra sinica	myricadiol
Ephedra sinica	sabinene
Ephedra sinica	cibarian
Ephedra sinica	(4s,5r) ephedroxane
Ephedra sinica	ephedrine
Ephedra sinica	d-norpseudoephedrine
Ephedra sinica	lauric acid
Ephedra sinica	limonene
Ephedra sinica	1-octanol
Ephedra sinica	phenethylamine
Ephedra sinica	nerolidol
Ephedra sinica	herbacetin-3-beta-d-(2-o-beta-d-glucopyra-nosidogluco-pyranoside)-8-beta-d-glucopyranoside
Ephedra sinica	carvacrol
Ephedra sinica	kaempferol-3-arabofuranoside
Ephedra sinica	3-methyl phenanthrene
Ephedra sinica	benzaldehyde
Ephedra sinica	xylene
Ephedra sinica	1-octen-3-ol
Ephedra sinica	methylpseudoephedrine
Ephedra sinica	herbacetin
Ephedra sinica	alpha-terpinolene
Ephedra sinica	vanillic acid
Ephedra sinica	octanoic acid
Ephedra sinica	m-xylene

Ephedra sinica	3-methoxyherbacetin
Ephedra sinica	11-methoxyhumantenine
Ephedra sinica	heneicosane
Ephedra sinica	hexanoic acid
Ephedra sinica	ethanol
Ephedra sinica	2,3,5,6-tetramethyl-pyrazine
Ephedra sinica	geraniol
Ephedra sinica	octadecane
Ephedra sinica	linalool oxide
Ephedra sinica	heptanoic acid
Ephedra sinica	methylacetophenone
Ephedra sinica	alpha-ionone
Ephedra sinica	ephedroxane
Ephedra sinica	cis-β -terpineol
Ephedra sinica	terpinen-4-ol
Ephedra sinica	apigenin
Ephedra sinica	hexanol
Ephedra sinica	octanol
Ephedra sinica	α -terpineol
Ephedra sinica	trans-β -terpineol
Zingiber officinale	(3r,5s)-3-acetoxy-5-hydroxy-1,7-bis(4-hy-droxy-3-methoxyphenyl)heptane
Zingiber officinale	α -cubebene
Zingiber officinale	[6]-gingerdione (enol form)
Zingiber officinale	[10]-gingediol
Zingiber officinale	[8]-gingediol
Zingiber officinale	[6]-gingediol
Zingiber officinale	[6]-gingediacetate
Zingiber officinale	camphor
Zingiber officinale	1-(4-hydroxy-3-methoxyphenyl)propan-1,2-diol
Zingiber officinale	d-limonene
Zingiber officinale	(5s)-5-acetoxy-1,7-bis(4-hydroxy-3-methoxy-phenyl)heptan-3-one
Zingiber officinale	1,5-epoxy-3-hydroxy-1-(4-hydroxy-3,5-dimethoxyphenyl)-7-(4-hydroxy-3-methoxy-phenyl)heptane
Zingiber officinale	camphene
Zingiber officinale	difengpin
Zingiber officinale	α -copaene
Zingiber officinale	α -curcumene
Zingiber officinale	zingiberol
Zingiber officinale	ethylisopropyl sulfide
Zingiber officinale	5-hydroxy-1-(4-hydroxy-3-methoxyphenyl)-7-(3,4-dihydroxyphenyl)heptan-3-one
Zingiber officinale	5-hydroxy-1-(4-hydroxy-3-methoxyphenyl)-7-(3,4-dihydroxy-5-methoxyphenyl)heptan-3-one
Zingiber officinale	isobrucine
Zingiber officinale	(e)-2-nonenal
Zingiber officinale	gingerenone a
Zingiber officinale	γ -terpinene
Zingiber officinale	eudesobovatol a

Zingiber officinale	beta-eudesmol
Zingiber officinale	(3r,5s)-3,5-dihydroxy-1-(4-hydroxy-3,5-dimethoxyphenyl)-7-(4-hydroxy-3-methoxyphenyl)heptane
Zingiber officinale	fenchyl alcohol
Zingiber officinale	(+-)-car-3-ene-2,5-dione
Zingiber officinale	fenfangjine a
Zingiber officinale	citral
Zingiber officinale	(e)-citral
Zingiber officinale	(z)-citral
Zingiber officinale	methylacetate
Zingiber officinale	9-ethoxy-aristololactam
Zingiber officinale	4-ethenyl-2,2,4-trimethyl-3-(1-methylethenyl)-cyclohexane-methanol
Zingiber officinale	citronellol
Zingiber officinale	citronellyl acetate
Zingiber officinale	6-hydroxy-2-[2-(4'-methoxyphenyl)-ethyl] chromone
Zingiber officinale	methyl a lly tetraslfide
Zingiber officinale	coprine
Zingiber officinale	alpha-bergamotene
Zingiber officinale	propionaldehyde
Zingiber officinale	cubebin
Zingiber officinale	1-(4-hydroxy-3-methoxyphenyl)-3,5-octane-diol
Zingiber officinale	4,8,12-trimethyl tridecanoic acid
Zingiber officinale	borneol
Zingiber officinale	methyl guaia-1(10),11-dien-15-carboxylate
Zingiber officinale	d-alpha-pipecoline
Zingiber officinale	Δ 3-menthene
Zingiber officinale	β -caryophyllene
Zingiber officinale	Δ 1(7)-menthene
Zingiber officinale	eugenol methyl ether
Zingiber officinale	β -farnesene
Zingiber officinale	β -bisabolene
Zingiber officinale	α -pinene
Zingiber officinale	3,5-diacetoxy-1-(4-hydroxy-3,5-dimethoxy-phenyl)-7-(4-hydroxy-3-methoxyphenyl)heptane
Zingiber officinale	p-menth-2-en-7-ol
Zingiber officinale	citropten
Zingiber officinale	methylglyoxal
Zingiber officinale	6-methylgingediol
Zingiber officinale	6-methylgingediacetate
Zingiber officinale	β -cis-ocimene
Zingiber officinale	8-geranyloxy psoralen
Zingiber officinale	octanoic acid
Zingiber officinale	6-acetyl gingerol
Zingiber officinale	1-(4-hydroxy-3-methoxyphenyl)-3,5-diacetoxy-octane
Zingiber officinale	4-methyl-1-(1-methylethyl)-3-cyclohexen-1-ol-acetate
Zingiber officinale	tricyclodehydroisohumulone
Zingiber officinale	pipecolicacid

Zingiber officinale	(z,z)-α -farnesene
Zingiber officinale	n-butylaldehyde
Zingiber officinale	shogasulfonicacid d
Zingiber officinale	farnesiferol a
Zingiber officinale	eugenone
Zingiber officinale	diethyl sulfide
Zingiber officinale	n-butyl allophanate
Zingiber officinale	α -bergamotene
Zingiber officinale	delta-elemene
Zingiber officinale	farnesol
Zingiber officinale	zonarene
Zingiber officinale	nonanoic acid
Zingiber officinale	campherenol
Zingiber officinale	terpinolene
Zingiber officinale	terpinyl acetate
Zingiber officinale	farnesyl acetate
Zingiber officinale	2-methyl-5-acetonyl-7-hydroxychromone
Zingiber officinale	beta-curcumene
Zingiber officinale	nerol
Zingiber officinale	2-nonanone
Zingiber officinale	nereistoxin
Zingiber officinale	delta7-menthene
Zingiber officinale	ethyllaurate
Zingiber officinale	methyl allyl sulfide
Zingiber officinale	p-cymene
Zingiber officinale	caranine
Zingiber officinale	3-caraneol
Zingiber officinale	2-caraneol
Zingiber officinale	hptaphylline
Zingiber officinale	linalool
Zingiber officinale	gingerdione
Zingiber officinale	beta-pinene
Zingiber officinale	cis-methyl isoeugenol
Zingiber officinale	methyl isobutyl ketone
Zingiber officinale	zingerone
Zingiber officinale	zingiberene
Zingiber officinale	10-gingerol
Zingiber officinale	4'-o-methylglabridin
Zingiber officinale	geraniin
Zingiber officinale	nonane
Zingiber officinale	gingerol
Zingiber officinale	acetylborneol
Zingiber officinale	paravallarine
Zingiber officinale	1-thujone
Zingiber officinale	thujene
Zingiber officinale	phellatin
Zingiber officinale	β -phellandrene

Zingiber officinale	propionic acid
Zingiber officinale	4-gingerol
Zingiber officinale	β -santalol
Zingiber officinale	santamarin
Zingiber officinale	cinerins
Zingiber officinale	isovaleraldehyde
Zingiber officinale	heptane
Zingiber officinale	ethyl acetate
Zingiber officinale	zygacine
Zingiber officinale	ethyl aldehyde
Zingiber officinale	2-heptanol
Zingiber officinale	isovaleric acid
Zingiber officinale	10-gingediol
Zingiber officinale	nervogenic acid
Zingiber officinale	8-gingediol
Zingiber officinale	6-gingediol
Zingiber officinale	1,3,3-trimethyltricyclo[2.2.1.0(2,6)]heptane
Zingiber officinale	6-paradol
Zingiber officinale	5-hydroxy-1-(3,4-dihydroxy-5-methoxyphenyl)-7-(4-hydroxy-3-methoxyphenyl)heptan-3-one
Zingiber officinale	12,13-di-acetoxyl-1,4,6,11-eudesmanetetol
Zingiber officinale	citraurin beta
Zingiber officinale	1-(1,5-dimethyl-4-hexenyl)-4-methyl benzene
Zingiber officinale	1,8-cineole
Zingiber officinale	safrole
Zingiber officinale	safynol
Zingiber officinale	cis-belta-terpineol
Zingiber officinale	(e)-8β ,17-epoxylabd-12-ene-15,16-dial
Zingiber officinale	shogasulfonicacid b
Zingiber officinale	2,5-dimethyl-7-hydroxy chromone
Zingiber officinale	delta-terpineol
Zingiber officinale	e-nerolidol
Zingiber officinale	z-nerolidol
Zingiber officinale	isoginkgetin
Zingiber officinale	beta-bisabolene
Zingiber officinale	isogingerenone b
Zingiber officinale	alpha-limonene
Zingiber officinale	i-linalool
Zingiber officinale	meso-3,5-diacetoxy-1,7-bis-(4-hydroxy-3-methoxyphenyl)heptane
Zingiber officinale	mesotrihydroxypiperidine
Zingiber officinale	alloaromadendrene
Zingiber officinale	terinen-4-ol
Zingiber officinale	elemol
Zingiber officinale	shyobunone
Zingiber officinale	shogasulfonicacid a
Zingiber officinale	shogasulfonicacid c
Zingiber officinale	γ -elemene

Zingiber officinale	β -elemene
Zingiber officinale	6-shogaol
Zingiber officinale	6-gingerol
Zingiber officinale	elenolide
Zingiber officinale	n-propanol
Zingiber officinale	shogaol
Zingiber officinale	dehydroglaucine
Zingiber officinale	10-dehydrogingerdione
Zingiber officinale	6-dehydrogingerdione
Zingiber officinale	alpha-fenchene
Zingiber officinale	propapyriogenin a2
Zingiber officinale	alpha-farnesene
Zingiber officinale	methyl-4-o-methylgallate
Zingiber officinale	(+)-1,5-epoxy-nor-ketoguaia-11-ene
Zingiber officinale	glyoxal
Zingiber officinale	alpha-cedrene
Zingiber officinale	cynanuriculoside a
Zingiber officinale	octanal
Zingiber officinale	gamma-camphorene
Zingiber officinale	aframodial
Zingiber officinale	1-(4-hydroxy-3-methoxyphenyl-2-[4-(omega-hydroxypropyl)-2-methoxyphenoxy]-propane-1,3-diol
Zingiber officinale	tricyclene
Zingiber officinale	ethylpropionate
Zingiber officinale	methyl hexadecanate
Zingiber officinale	4-ethylresorcinol
Zingiber officinale	n-nonane
Zingiber officinale	2-nonanol
Zingiber officinale	n-nonanol
Zingiber officinale	4-gingesulfonicacid
Zingiber officinale	6-gingesulfonicacid
Zingiber officinale	glypallichalcone
Zingiber officinale	[6]-gingerol
Zingiber officinale	zingiberone
Zingiber officinale	gingerenone b
Zingiber officinale	gingerenone c
Zingiber officinale	(3s,5s)-3,5-diacetoxy-1,7-bis(4-hydroxy-3-methoxyphenyl)heptane
Zingiber officinale	[4]-gingerol
Zingiber officinale	elemicin
Zingiber officinale	β -cedrene
Zingiber officinale	geraniol
Zingiber officinale	geranyl acetate
Zingiber officinale	bata-caryophyllene
Zingiber officinale	galanolactone
Zingiber officinale	galanthamine
Zingiber officinale	d-isoborneol
Zingiber officinale	l-isoborneol

Zingiber officinale	perilla ketone
Zingiber officinale	perilal
Zingiber officinale	terpinen-4-ol
Zingiber officinale	carene-3
Zingiber officinale	n-octane
Zingiber officinale	β -fenchene
Zingiber officinale	α -terpineol
Atractylodes macrocephala	butenolide b
Atractylodes macrocephala	beta-eudesmol
Atractylodes macrocephala	12(alpha-methyl butyryl)-14-acetyl-2e,8z,10e-atractylentriol
Atractylodes macrocephala	14(alpha-methyl butyryl)-2e,8e,10e-atractylentriol
Atractylodes macrocephala	14(alpha-methyl butyryl)-2e,8z,10e-atractylentriol
Atractylodes macrocephala	2alpha-(alpha-methylbutyrul)-oxy-5alpha,7belta,9alpha,10belta-tetraacetoxy-4(20),11-taxadiene
Atractylodes macrocephala	atracty lentrid
Atractylodes macrocephala	atractyenolide
Atractylodes macrocephala	atractylentriol
Atractylodes macrocephala	atractylodin
Atractylodes macrocephala	atractylenolide i
Atractylodes macrocephala	atractylenolide iii
Atractylodes macrocephala	(+)-eudesma-4(15),7(11)-dien-8-one
Atractylodes macrocephala	butenolide a
Atractylodes macrocephala	4-ethoxycarbonyl-2-quinolone
Atractylodes macrocephala	hinesol
Atractylodes macrocephala	8β -ethoxy atractylenolide iii
Atractylodes macrocephala	atractylenolide
Atractylodes macrocephala	atractylon
Atractylodes macrocephala	12(alpha-methyl butyryl)-14-acetyl-2e,8z,10e-atractylentriol
Atractylodes macrocephala	12-(α -methyl butyryl)-14-acetyl-2 e,8 e,10 e-atractylentriol
Atractylodes macrocephala	atractylone
Atractylodes macrocephala	juniper camphor
Atractylodes macrocephala	jurubine
Saposhnikovia divaricata	1-hexanol
Saposhnikovia divaricata	3'(s)-(−)-hamaudol
Saposhnikovia divaricata	hancinol
Saposhnikovia divaricata	cuparene
Saposhnikovia divaricata	3-methyl-3-butenone
Saposhnikovia divaricata	deltoin
Saposhnikovia divaricata	panaxytriol
Saposhnikovia divaricata	suffruticoside a
Saposhnikovia divaricata	phenethyl alcohol
Saposhnikovia divaricata	n-octane
Saposhnikovia divaricata	phellopterin
Saposhnikovia divaricata	4'-o-β -glucopyranosyl-5-o-methylvisamminol
Saposhnikovia divaricata	panaxydol
Saposhnikovia divaricata	deltonin (e)
Saposhnikovia divaricata	prim-o-glucosylcimifugin

Saposhnikovia divaricata	alpha-cuparenol
Saposhnikovia divaricata	cimicifugoside
Saposhnikovia divaricata	bergapten
Saposhnikovia divaricata	fallacinol
Saposhnikovia divaricata	psoralen
Saposhnikovia divaricata	dacursin
Saposhnikovia divaricata	tetradecane
Saposhnikovia divaricata	daucosterol
Saposhnikovia divaricata	imperatorin
Saposhnikovia divaricata	naphthazarin
Saposhnikovia divaricata	naphthalene
Saposhnikovia divaricata	pentanal
Saposhnikovia divaricata	3'-o-acetylhamaudol
Saposhnikovia divaricata	eudesobovatol a
Saposhnikovia divaricata	(3's)-hydroxydeltoin
Saposhnikovia divaricata	octanal
Saposhnikovia divaricata	beta-eudesmol
Saposhnikovia divaricata	3'-o-angeloylhamaudol
Saposhnikovia divaricata	5-o-methylvisamminol
Saposhnikovia divaricata	lignocericacid
Saposhnikovia divaricata	gamma-sitosterol
Saposhnikovia divaricata	ledebouriellol
Saposhnikovia divaricata	2-(beta-d-glucopyranosyloxy)-8-hydroxy-1-methoxy-3-methyl-9,10-anthraquinon e
Saposhnikovia divaricata	α -pinene
Saposhnikovia divaricata	2-o-methyl-d-xylose
Saposhnikovia divaricata	1,8-heptadecadiene-4,6-diyne-3,10-diol
Saposhnikovia divaricata	heptadecane
Saposhnikovia divaricata	heptadeca-1-en-4,6-diyne-3,9-diol
Saposhnikovia divaricata	lignoceric acid
Saposhnikovia divaricata	primetin
Saposhnikovia divaricata	2-methylbut-3-en-2-ol
Saposhnikovia divaricata	pentanic acid
Saposhnikovia divaricata	sucrose
Saposhnikovia divaricata	divaricatol
Saposhnikovia divaricata	β -sitosterol
Saposhnikovia divaricata	beta-pinene
Saposhnikovia divaricata	chromone
Saposhnikovia divaricata	cis-falcarindiol
Saposhnikovia divaricata	pentanol
Saposhnikovia divaricata	dauricine
Saposhnikovia divaricata	1-octen-3-ol
Saposhnikovia divaricata	4-pentenyl isothiocyanate
Saposhnikovia divaricata	3-o-angeloyl-hamaudol
Saposhnikovia divaricata	panaxynol
Saposhnikovia divaricata	beta-bisabolene
Saposhnikovia divaricata	(e)-2-hexenal

Saposhnikovia divaricata	ledol
Saposhnikovia divaricata	imperialine
Saposhnikovia divaricata	prim-o-gluco sylcimifogin
Saposhnikovia divaricata	β -bisabolene
Saposhnikovia divaricata	hexanal
Saposhnikovia divaricata	hexanol
Saposhnikovia divaricata	octanol
Saposhnikovia divaricata	3-octanol
Saposhnikovia divaricata	7-octen-4-ol
Saposhnikovia divaricata	heptadeca-1-en-4,6-dihy-3,9-diol
Cinnamomum cassia	cinnamaldehyde
Cinnamomum cassia	anhydrocinnzeylanine
Cinnamomum cassia	beta-sitoterol
Cinnamomum cassia	2-methoxycinnamaldehyde
Cinnamomum cassia	cinnamicacid
Cinnamomum cassia	camphor
Cinnamomum cassia	tetradecanal
Cinnamomum cassia	dihydromelilotoside
Cinnamomum cassia	protocatechuic acid
Cinnamomum cassia	3,4-dihydroxybenzoicacid
Cinnamomum cassia	nerolidol
Cinnamomum cassia	protocatechuic acid-3-glucoside
Cinnamomum cassia	(+-)-2,3-dihydro-2-(1-methylethenyl)-5-benzofurancarboxylic acid methyl ester
Cinnamomum cassia	melilotocarpan a
Cinnamomum cassia	procurcumenol
Cinnamomum cassia	meliloticacid
Cinnamomum cassia	benzaldehyde
Cinnamomum cassia	coumarin
Cinnamomum cassia	cinnamicalcohol
Cinnamomum cassia	5-cinnamoyl-9-o-acetylphototaxicin i
Cinnamomum cassia	trans-cinnamic acid
Cinnamomum cassia	cinnamic aldehyde
Cinnamomum cassia	gamma-sitosterol
Cinnamomum cassia	farnesol
Cinnamomum cassia	3-o-p-hydroxy-trans-cinnamoylmaslinic acid
Cinnamomum cassia	coumarinic acid
Cinnamomum cassia	styrene
Cinnamomum cassia	β -sitosterol
Cinnamomum cassia	2'-hydroxycinnamaldehyde
Cinnamomum cassia	anhydrocinnzeylanol
Cinnamomum cassia	proanthocyanidin b2
Cinnamomum cassia	anethole
Cinnamomum cassia	3-(4-hdroxyphenyl)-trans-propenoic acid-2,3-dihydroxypropyl ester
Cinnamomum cassia	acetic acid
Paeonia albiflora	paeonol
Paeonia albiflora	1,2,3-tri-o-galloyl-β -d-glucose
Paeonia albiflora	1,2,6-tri-o-galloyl-β -d-glucose

Paeonia albiflora	1,3,6-trigalloyl-β -d-glucose
Paeonia albiflora	d-catechin
Paeonia albiflora	paeonianin a
Paeonia albiflora	paeonianiin e
Paeonia albiflora	palbinone
Paeonia albiflora	1,2,3,4,6-pentagalloylglucose
Paeonia albiflora	catechin
Paeonia albiflora	(+)-catechin
Paeonia albiflora	4-o-galloyl quinicacid
Paeonia albiflora	3-o-galloyl quinicacid
Paeonia albiflora	pyrethrin ii
Paeonia albiflora	benzoic acid
Paeonia albiflora	albiflorin
Paeonia albiflora	casuariin
Paeonia albiflora	benzoicacid
Paeonia albiflora	epigallocatechin
Paeonia albiflora	13-methyl tetradecanoicacid
Paeonia albiflora	eugeniin
Paeonia albiflora	gallotannin
Paeonia albiflora	pyrethrin i
Paeonia albiflora	beta-sitosterol
Paeonia albiflora	1, 2, 3, 4, 6-pentagalloylglucose
Paeonia albiflora	casuarictin
Paeonia albiflora	benzoylpaeoniflorin
Paeonia albiflora	(-)-catechin
Paeonia albiflora	pedunculagin
Paeonia albiflora	1-o-galloyl-glucose
Paeonia albiflora	paeoniflorigenone
Paeonia albiflora	tellimagrandin i
Paeonia albiflora	1,2,3,6-tetra-o-galloyl-β -d-glucose
Paeonia albiflora	paeonianin b
Paeonia albiflora	paeonianin c
Paeonia albiflora	paeonianin d
Paeonia albiflora	2,3-o-(s)-hexahydroxydiphenoyl-d-gluco-pyranose
Paeonia albiflora	paeoniflorin
Paeonia albiflora	paeonilactone a
Paeonia albiflora	paeonilactone b
Paeonia albiflora	paeonilactone c
Paeonia albiflora	(z)-(1s,5r)-beta-pinen-1-oxy-beta-vicianoside
Paeonia albiflora	gallicacid
Paeonia albiflora	phenol
Paeonia albiflora	β -sitosterol
Paeonia albiflora	gallocatechin
Paeonia albiflora	oxypaeoniflorin
Paeonia albiflora	5-desgalloylstachyurin
Paeonia albiflora	albiflorinr1
Paeonia albiflora	paeonin

Paeonia albiflora	lactiflorin
Paeonia albiflora	peonin
Paeonia albiflora	strictinin
Paeonia albiflora	acetic acid
Anemarrhena asphodeloides	sarsasapogenin
Anemarrhena asphodeloides	monomethyl-cis-hinokiresinol
Anemarrhena asphodeloides	monomethyl lithospermate
Anemarrhena asphodeloides	chrysanthemaxanthin
Anemarrhena asphodeloides	timosaponin a- i, a- ii, a- iii, a- iv, b- i & b- ii
Anemarrhena asphodeloides	neogitogenin
Anemarrhena asphodeloides	macrostemonoside f
Anemarrhena asphodeloides	smilagenin-3-o-[β -d-glucopyranosyl-(1→2)]-β -d-mannopyranoside
Anemarrhena asphodeloides	timosaponin a1
Anemarrhena asphodeloides	timosaponin a3
Anemarrhena asphodeloides	isomangiferin
Anemarrhena asphodeloides	ginkgetin
Anemarrhena asphodeloides	4-o-beta-d-glucopyranosyl methyl gallate
Anemarrhena asphodeloides	timosaponin a-iii
Anemarrhena asphodeloides	anemarrhenasaponin-ia
Anemarrhena asphodeloides	timobiose
Anemarrhena asphodeloides	nicotinamide
Anemarrhena asphodeloides	mangiferolic acid
Anemarrhena asphodeloides	(25r,s)-5alpha-spirostane-2alpha,3beta-diol 3-o-[o-beta-d-glucopyranosyl-(1-2)-o-beta-d-glucopyranosyl-(1-4)-beta-d-galacto pyranoside]
Anemarrhena asphodeloides	desglucolanatigonin ii
Anemarrhena asphodeloides	(5 β ,25s)-spirostan-3 β ,15 α ,23 α -diol-3-o-d-glu-copyranosyl-(1 → 2)- β -d-galactopyranoside
Anemarrhena asphodeloides	markogenin-3-o-beta-d-glucopyranosyl-(1-2)-beta-d-galactopyranoside
Anemarrhena asphodeloides	markogenin
Anemarrhena asphodeloides	markogenin3-o-β -d-glucopyranosyl-(1→2)-β -d-galactopyranoside
Anemarrhena asphodeloides	ginkgolic acid
Anemarrhena asphodeloides	anemarrhenasaponin-i
Anemarrhena asphodeloides	degalactotigonin
Anemarrhena asphodeloides	nicotine
Anemarrhena asphodeloides	anemarans a, b, c, d
Anemarrhena asphodeloides	hippeastrine
Anemarrhena asphodeloides	cis-hinokiresinol
Anemarrhena asphodeloides	isomangiferolic acid
Anemarrhena asphodeloides	anemarsaponin g
Anemarrhena asphodeloides	anemarsaponin f
Anemarrhena asphodeloides	anemarsaponin e
Anemarrhena asphodeloides	anemarsaponin c
Anemarrhena asphodeloides	anemarsaponin b
Anemarrhena asphodeloides	anemarrhenasaponin ia
Anemarrhena asphodeloides	anemarrhenasaponin i
Anemarrhena asphodeloides	sarsasapogenin 3-o-4-rhamnosyl-sophoroside

Anemarrhena asphodeloides	choline
Anemarrhena asphodeloides	tingenone
Anemarrhena asphodeloides	sarsasapogenin-3-o-beta-d-glucopyranosyl(1->2)-beta-galactopyranoside
Anemarrhena asphodeloides	smilagenone
Anemarrhena asphodeloides	xilingsaponin b
Anemarrhena asphodeloides	mangiferin
Anemarrhena asphodeloides	neoglucobrassicin
Anemarrhena asphodeloides	12-o-nicotinoylisolineolone
Anemarrhena asphodeloides	smilagenin
Anemarrhena asphodeloides	nicotinicacid
Anemarrhena asphodeloides	makrkogenin-3-o-beta-d-glucopyranosyl-(1->2)-beta-d-galactopyranoside
Anemarrhena asphodeloides	(5 β ,25s)-spirostan-3 β ,15 α ,23 α -triol-3-o-d-glu-copyranosyl-(1 → 2)- β -d-galactopyranoside
Anemarrhena asphodeloides	cthd0233276-21
Anemarrhena asphodeloides	spongesterol
Anemarrhena asphodeloides	pentacosyl vinyl ester
Anemarrhena asphodeloides	manguferin
Anemarrhena asphodeloides	smilagenin-3-o[beta-d-glucopyranosyl-(1-2)]-beta-d-mannopyranoside
Anemarrhena asphodeloides	p-hydroxyphenyl crotonicacid
Anemarrhena asphodeloides	(25s)-26-o-β -d-glucopyranosyl-22-hydroxy-5β -furostane-3β ,26-diol 3-o-β -d-glucopy-ranosyl-(1→2)-o-β -d-galactopyranoside
Anemarrhena asphodeloides	xuelianlacton e
Anemarrhena asphodeloides	marmesin
Anemarrhena asphodeloides	timosaponin a-1

Supplement Table S2 GO enrichment analysis result of GSZ formula. Gene ontology analysis was implemented by Cytoscape 3.2.1 with ClueGo plugin. Enriched GOterms and pathways were defined as those with adjust P-value <0.05.

**GO Term**	**GO Levels**	**Nr. Genes**	**% Associated Genes**	**Term P-value**
leukocyte differentiation	[4]	79	19.458128	2.62E-04
myeloid cell differentiation	[4]	64	20.447285	2.75E-04
positive regulation of leukocyte migration	[2, 3]	23	28.04878	4.07E-04
regulation of myeloid leukocyte differentiation	[3, 6]	25	26.31579	6.63E-04
positive regulation of lymphocyte activation	[3, 4]	51	20.564516	0.001110119
regulation of lymphocyte proliferation	[4]	38	22.093023	0.001280333
regulation of osteoclast differentiation	[4, 7]	16	30.188679	0.001330658
positive regulation of myeloid cell differentiation	[3, 5, 6]	20	27.027027	0.001610784
positive regulation of leukocyte chemotaxis	[3, 4]	17	28.333334	0.002058184
positive regulation of lymphocyte proliferation	[4, 5]	27	23.68421	0.002310227
cellular response to interferon-gamma	[4]	25	24.271845	0.002333111
positive regulation of leukocyte activation	[2, 3]	53	19.702602	0.002473024
regulation of lymphocyte activation	[3]	66	18.75	0.002682818
hemopoiesis	[3]	108	17.11569	0.002914032
response to interferon-gamma	[3]	28	22.764227	0.003582403
regulation of production of molecular mediator of immune response	[2, 3]	20	24.691359	0.005111878
lymphocyte proliferation	[3]	45	19.48052	0.006683075
regulation of cytokine production involved in immune response	[3, 4]	14	27.450981	0.0068537
regulation of complement activation	[3, 4, 5,	10	31.25	0.008168445
positive regulation of myeloid leukocyte differentiation	[4, 6, 7]	12	27.906977	0.010450269
regulation of leukocyte chemotaxis	[3]	17	23.943663	0.01309351
positive regulation of T cell activation	[4, 5]	37	19.473684	0.013763082
macrophage activation	[3]	13	26	0.014522206
regulation of immunoglobulin production	[3, 4]	11	27.5	0.015672519
myeloid leukocyte mediated immunity	[3]	14	25	0.016145371
positive regulation of immune effector process	[2, 3]	27	20.610687	0.016616912
T cell activation	[3]	70	17.156862	0.018627138
positive regulation of B cell activation	[4, 5]	15	23.809525	0.020218682
regulation of T cell activation	[4]	48	18.113207	0.020397456
T cell proliferation	[4]	29	19.594595	0.025805197
lymphocyte costimulation	[1, 4, 5]	18	21.95122	0.02593301
B cell activation	[3]	39	18.396227	0.028224325
B cell activation involved in immune response	[4, 5]	12	24.489796	0.02926758
granulocyte migration	[3]	18	21.17647	0.036223979
regulation of humoral immune response	[3]	12	23.529411	0.039086589
granulocyte chemotaxis	[3, 4]	17	20.987654	0.044426637
B cell proliferation	[4]	18	20.454546	0.049243673
pattern recognition receptor signaling pathway	[4, 6, 7]	32	18.181818	0.051670824
innate immune response-activating signal transduction	[3, 5, 6]	32	17.877094	0.063011232

regulation of B cell activation	[4]	19	19.58763	0.064443742
activation of innate immune response	[2, 4, 5]	33	17.741936	0.065141854
neutrophil chemotaxis	[4, 5]	13	20.967741	0.073816625
neutrophil migration	[4]	13	20.967741	0.073816625
positive regulation of leukocyte differentiation	[3, 5, 6]	21	18.75	0.07929956
regulation of leukocyte mediated immunity	[3]	22	17.741936	0.117936383
regulation of innate immune response	[3]	45	16.18705	0.122565024
lymphocyte activation involved in immune response	[3, 4]	21	17.355371	0.14724319
positive regulation of innate immune response	[3, 4]	35	16.203703	0.158803532
somatic diversification of immune receptors via germline recombination within a single locus	[2, 3]	11	18.965517	0.163161453
T cell differentiation	[4, 6]	30	16.216217	0.181040371
lymphocyte differentiation	[3, 5]	43	15.693431	0.181980394
somatic diversification of immunoglobulins	[2, 3]	10	18.867924	0.182524584
positive regulation of leukocyte mediated immunity	[3, 4]	12	18.181818	0.18620082
leukocyte activation involved in immune response	[2, 3]	29	16.022099	0.205292316
erythrocyte differentiation	[3, 5]	18	16.82243	0.207469395
negative regulation of lymphocyte proliferation	[4, 5]	10	17.857143	0.232143454
negative regulation of leukocyte activation	[2, 3]	20	16.260162	0.237696445
B cell differentiation	[4, 6]	17	16.346153	0.253927364
positive regulation of adaptive immune response	[3, 4]	11	17.1875	0.256802912
regulation of T cell differentiation	[4, 5, 7]	15	15.789474	0.320337425
positive regulation of adaptive immune response based on somatic recombination of immune receptors built from immunoglobulin superfamily domains	[4, 5]	10	16.393442	0.324055278
regulation of adaptive immune response	[3]	18	15.5172415	0.324297191
negative regulation of lymphocyte activation	[3, 4]	16	15.53398	0.336860504
negative regulation of leukocyte differentiation	[3, 5, 6]	11	15.942029	0.345940478
positive regulation of lymphocyte mediated immunity	[4, 5]	10	15.873015	0.362879004
regulation of lymphocyte mediated immunity	[4]	15	15.306123	0.367088115
hematopoietic progenitor cell differentiation	[4]	13	15.294118	0.383440045
negative regulation of T cell activation	[4, 5]	11	15.068493	0.420676973
negative regulation of immune response	[2, 3]	10	15.151515	0.422015421
alpha-beta T cell activation	[4]	14	14.43299	0.466089097
T cell mediated immunity	[4]	11	14.473684	0.476887152
regulation of adaptive immune response based on somatic recombination of immune receptors built from immunoglobulin superfamily domains	[4]	15	14.018692	0.510532189
regulation of lymphocyte differentiation	[3, 4, 6]	17	13.934426	0.516671269
T cell activation involved in immune response	[4, 5]	10	13.888889	0.538687456
antigen receptor-mediated signaling pathway	[4, 6, 7]	21	13.636364	0.553628477
negative regulation of myeloid cell differentiation	[3, 5, 6]	10	13.157895	0.611682214
positive regulation of immune response	[2, 3]	87	13.384615	0.644244342
regulation of defense response to virus	[3]	10	12.345679	0.694169113

immune response-regulating signaling pathway	[3]	80	12.924071	0.77349101
activation of immune response	[1, 3, 4]	72	12.788632	0.79332221
immune response-regulating cell surface receptor signaling [illegible]	[4]	60	12.42236	0.845445042
adaptive immune response based on somatic recombination of [illegible]	[3]	40	11.594203	0.9114423
B cell mediated immunity	[4]	26	10.878661	0.930387853
immune response-activating signal transduction	[2, 4, 5]	57	11.309524	0.970348188

Supplementary Table S3 Pathway enrichment analysis result of GZS formula targets. DAVID Bioinformatics Resources 6.7 (http://david.abcc.ncifcrf.gov/) was used to carry out pathway enrichment for the 1,327 genes targeted by GSZ decoction. Totally 57 pathways were enriched.

pathway	Genes number	P-value	Class
Neuroactive ligand-receptor interaction	139	1.80E-45	Environmental Information Processing
Calcium signaling pathway	73	4.10E-15	Environmental Information Processing
Glycolysis / Gluconeogenesis	27	7.90E-07	Metabolism- Carbohydrate metabolism
beta-Alanine metabolism	15	9.00E-07	Metabolism- Carbohydrate metabolism
Pyruvate metabolism	22	1.70E-07	Metabolism-Carbohydrate metabolism
Drug metabolism	32	9.30E-10	Metabolism- Xenobiotics biodegradation and metabolism
Drug metabolism	22	8.30E-07	Metabolism- Xenobiotics biodegradation and metabolism
Metabolism of xenobiotics by cytochrome P450	30	8.90E-09	Metabolism-Xenobiotics biodegradation and metabolism
Tyrosine metabolism	22	1.30E-06	Metabolism-Amino acid metabolism
Linoleic acid metabolism	27	9.20E-19	Metabolism-Lipid metabolism
Arachidonic acid metabolism	36	9.50E-15	Metabolism-Lipid metabolism
Ether lipid metabolism	24	9.10E-11	Metabolism-Lipid metabolism
alpha-Linolenic acid metabolism	16	8.50E-10	Metabolism-Lipid metabolism
Glycerophospholipid metabolism	31	6.80E-08	Metabolism-Lipid metabolism
Retinol metabolism	27	5.80E-08	Metabolism-Metabolism of cofactors and vitamins
Fatty acid metabolism	24	4.20E-09	Metabolism-Overview
PPAR signaling pathway	42	6.30E-16	Organismal Systems-Endocrine system
Adipocytokine signaling pathway	35	8.30E-11	Organismal Systems-Endocrine system
GnRH signaling pathway	39	8.60E-08	Organismal Systems-Endocrine system
Fc epsilon RI signaling pathway	30	7.90E-06	Organismal Systems-Immune system
Vascular smooth muscle contraction	38	1.10E-05	Organismal Systems-Circulatory system
Apoptosis	31	3.10E-05	Cellular Processes-Cell growth and death
VEGF signaling pathway	28	3.20E-05	Environmental Information Processing-Signal transduction
Bladder cancer	19	4.80E-05	Human Diseases-cancers
Complement and coagulation cascades	26	5.50E-05	Organismal Systems-Immune system
Tryptophan metabolism	18	8.90E-05	Metabolism-Amino acid metabolism
Long-term depression	25	1.60E-04	Organismal Systems-Nervous system
Base excision repair	16	2.10E-04	Metabolism-Genetic Information Processing
Histidine metabolism	14	3.20E-04	Metabolism-Amino acid metabolism
Prostate cancer	29	3.40E-04	Human Diseases-cancers
Amyotrophic lateral sclerosis (ALS)	20	5.00E-04	Human Diseases-Neurodegenerative diseases
Prion diseases	15	7.80E-04	Human Diseases-Neurodegenerative diseases
Propanoate metabolism	14	1.00E-03	Metabolism-Carbohydrate metabolism
NOD-like receptor signaling pathway	21	1.60E-03	Organismal Systems- Immune system

Type II diabetes mellitus	17	2.50E-03	Human Diseases-Endocrine and metabolic diseases
Arginine and proline metabolism	18	3.80E-03	Metabolism-Amino acid metabolism
Pancreatic cancer	22	5.00E-03	Human Diseases-cancers
Steroid hormone biosynthesis	16	5.40E-03	Metabolism-Lipid metabolism
Phenylalanine metabolism	10	5.70E-03	Metabolism-Amino acid metabolism
ErbB signaling pathway	25	6.10E-03	Environmental Information Processing-Signal transduction
Insulin signaling pathway	35	6.10E-03	Organismal Systems-Endocrine system
Pathways in cancer	72	7.60E-03	Human Diseases-Cancers: Overview
Methane metabolism	5	8.40E-03	Metabolism-Energy metabolism
Glioma	19	1.10E-02	Human Diseases-cancers
Nicotinate and nicotinamide metabolism	10	1.10E-02	Metabolism-Metabolism of cofactors and vitamins
T cell receptor signaling pathway	28	1.50E-02	Organismal Systems-Immune system
Caffeine metabolism	5	1.70E-02	Metabolism-Biosynthesis of other secondary metabolites
Butanoate metabolism	12	1.70E-02	Metabolism-Carbohydrate metabolism
Neurotrophin signaling pathway	31	1.70E-02	Organismal Systems-Nervous system
Toll-like receptor signaling pathway	26	2.10E-02	Organismal Systems- Immune system
Glycine, serine and threonine metabolism	11	2.30E-02	Metabolism-Amino acid metabolism
Non-small cell lung cancer	16	2.50E-02	Human Diseases-cancers
MAPK signaling pathway	57	3.00E-02	Environmental Information Processing-Signal transduction
Small cell lung cancer	22	3.00E-02	Human Diseases-cancers
Starch and sucrose metabolism	13	3.50E-02	Metabolism-Carbohydrate metabolism
One carbon pool by folate	7	3.60E-02	Metabolism-Metabolism of cofactors and vitamins
Endometrial cancer	15	3.90E-02	Human Diseases-cancers

Supplementary Table S4 The list of drugs for RA treatment and drug targets. 79 targets of FDA 30 approved RA drugs were retrieved from DrugBank 4.0 (http://www.drugbank.ca).

**Drug name**	**Target name**	**Target ID**
Etanercept	Tumor necrosis factor	7124
Etanercept	Tumor necrosis factor receptor superfamily member 1B	7133
Etanercept	High affinity immunoglobulin gamma Fc receptor I	2209
Etanercept	High affinity immunoglobulin gamma Fc receptor I	100132417
Etanercept	Low affinity immunoglobulin gamma Fc region receptor III-A	2214
Etanercept	Low affinity immunoglobulin gamma Fc region receptor III-A	2215
Etanercept	Low affinity immunoglobulin gamma Fc region receptor II-a	2212
Etanercept	Low affinity immunoglobulin gamma Fc region receptor II-a	9103
Etanercept	Low affinity immunoglobulin gamma Fc region receptor II-b	2213
Etanercept	Low affinity immunoglobulin gamma Fc region receptor II-b	9103
Etanercept	Low affinity immunoglobulin gamma Fc region receptor II-c	9103
Etanercept	Lymphotoxin-alpha	4049
Etanercept	Low affinity immunoglobulin gamma Fc region receptor III-B	2215
Etanercept	Low affinity immunoglobulin gamma Fc region receptor III-B	2214
Etanercept	Complement C1s subcomponent	716
Etanercept	Complement C1r subcomponent	715
Etanercept	Complement C1q subcomponent subunit A	712
Etanercept	Complement C1q subcomponent subunit B	713
Etanercept	Complement C1q subcomponent subunit C	714
Intravenous Immunoglobulin	High affinity immunoglobulin gamma Fc receptor I	2209
Intravenous Immunoglobulin	High affinity immunoglobulin gamma Fc receptor I	100132417
Intravenous Immunoglobulin	High affinity immunoglobulin gamma Fc receptor IB	2210
Intravenous Immunoglobulin	Low affinity immunoglobulin gamma Fc region receptor II-a	2212
Intravenous Immunoglobulin	Low affinity immunoglobulin gamma Fc region receptor II-a	9103
Intravenous Immunoglobulin	Low affinity immunoglobulin gamma Fc region receptor II-b	2213
Intravenous Immunoglobulin	Low affinity immunoglobulin gamma Fc region receptor II-b	9103
Intravenous Immunoglobulin	Low affinity immunoglobulin gamma Fc region receptor II-c	9103
Intravenous Immunoglobulin	Low affinity immunoglobulin gamma Fc region receptor III-A	2214
Intravenous Immunoglobulin	Low affinity immunoglobulin gamma Fc region receptor III-A	2215
Intravenous Immunoglobulin	Low affinity immunoglobulin gamma Fc region receptor III-B	2215

Intravenous Immunoglobulin	Low affinity immunoglobulin gamma Fc region receptor III-B	2214
Intravenous Immunoglobulin	Complement C3	718
Intravenous Immunoglobulin	Complement C4-A	720
Intravenous Immunoglobulin	Complement C4-A	721
Intravenous Immunoglobulin	Complement C4-B	721
Intravenous Immunoglobulin	Complement C4-B	100293534
Intravenous Immunoglobulin	Complement C5	727
Adalimumab	Tumor necrosis factor	7124
Adalimumab	Low affinity immunoglobulin gamma Fc region receptor III-B	2215
Adalimumab	Low affinity immunoglobulin gamma Fc region receptor III-B	2214
Adalimumab	Complement C1r subcomponent	715
Adalimumab	Complement C1q subcomponent subunit A	712
Adalimumab	Complement C1q subcomponent subunit B	713
Adalimumab	Complement C1q subcomponent subunit C	714
Adalimumab	Low affinity immunoglobulin gamma Fc region receptor III-A	2214
Adalimumab	Low affinity immunoglobulin gamma Fc region receptor III-A	2215
Adalimumab	Complement C1s subcomponent	716
Adalimumab	High affinity immunoglobulin gamma Fc receptor I	2209
Adalimumab	High affinity immunoglobulin gamma Fc receptor I	100132417
Adalimumab	Low affinity immunoglobulin gamma Fc region receptor II-a	2212
Adalimumab	Low affinity immunoglobulin gamma Fc region receptor II-a	9103
Adalimumab	Low affinity immunoglobulin gamma Fc region receptor II-b	2213
Adalimumab	Low affinity immunoglobulin gamma Fc region receptor II-b	9103
Adalimumab	Low affinity immunoglobulin gamma Fc region receptor II-c	9103
Infliximab	Tumor necrosis factor	7124
Rituximab	Low affinity immunoglobulin gamma Fc region receptor III-B	2215
Rituximab	Low affinity immunoglobulin gamma Fc region receptor III-B	2214
Rituximab	Complement C1r subcomponent	715
Rituximab	Complement C1q subcomponent subunit A	712
Rituximab	Complement C1q subcomponent subunit B	713
Rituximab	Complement C1q subcomponent subunit C	714
Rituximab	Low affinity immunoglobulin gamma Fc region receptor III-A	2214
Rituximab	Low affinity immunoglobulin gamma Fc region receptor III-A	2215

Rituximab	Complement C1s subcomponent	716
Rituximab	High affinity immunoglobulin gamma Fc receptor I	2209
Rituximab	High affinity immunoglobulin gamma Fc receptor I	100132417
Rituximab	Low affinity immunoglobulin gamma Fc region receptor II-a	2212
Rituximab	Low affinity immunoglobulin gamma Fc region receptor II-a	9103
Rituximab	Low affinity immunoglobulin gamma Fc region receptor II-b	2213
Rituximab	Low affinity immunoglobulin gamma Fc region receptor II-b	9103
Rituximab	Low affinity immunoglobulin gamma Fc region receptor II-c	9103
Rituximab	B-lymphocyte antigen CD20	2206
Rituximab	B-lymphocyte antigen CD20	54474
Cyclosporine	Calcium signal-modulating cyclophilin ligand	819
Cyclosporine	Calcineurin subunit B type 2	5535
Cyclosporine	Peptidyl-prolyl cis-trans isomerase A	5478
Cyclosporine	"Peptidyl-prolyl cis-trans isomerase F, mitochondrial"	10105
L-Histidine	Histidine decarboxylase	51696
L-Histidine	"Histidine--tRNA ligase, cytoplasmic"	3035
L-Histidine	"Histidine--tRNA ligase, cytoplasmic"	9146
L-Histidine	Sodium-coupled neutral amino acid transporter 3	10991
L-Histidine	Histidine ammonia-lyase	3034
L-Histidine	Histidine ammonia-lyase	57016
L-Cystine	Cystine/glutamate transporter	23657
L-Cystine	Cystinosin	1497
L-Cystine	"B(0,+)-type amino acid transporter 1"	11136
L-Cystine	Neutral and basic amino acid transport protein rBAT	6519
Vitamin E	SEC14-like protein 4	284904
Vitamin E	SEC14-like protein 3	266629
Vitamin E	SEC14-like protein 2	23541
Vitamin E	Nuclear receptor subfamily 1 group I member 2	8856
Vitamin E	Protein kinase C beta type	5579
Vitamin E	Arachidonate 5-lipoxygenase	240
Vitamin E	Protein kinase C alpha type	5578
Vitamin E	Diacylglycerol kinase alpha	1606
Vitamin E	Diacylglycerol kinase alpha	1609
Vitamin E	Serine/threonine-protein phosphatase 2A catalytic subunit beta isoform	5516
Vitamin E	Serine/threonine-protein phosphatase 2A catalytic subunit alpha isoform	5515
Indomethacin	Prostaglandin G/H synthase 1	5742
Indomethacin	Prostaglandin G/H synthase 2	5743
Indomethacin	"Phospholipase A2, membrane associated"	5320
Indomethacin	"Phospholipase A2, membrane associated"	729330
Indomethacin	Prostaglandin reductase 2	145482
Indomethacin	Peroxisome proliferator-activated receptor gamma	5468
Indomethacin	Lactoylglutathione lyase	2739
Indomethacin	Prostaglandin D2 receptor 2	11251
Indomethacin	Peroxisome proliferator-activated receptor alpha	5465
Nabumetone	Prostaglandin G/H synthase 2	5743

Nabumetone	Prostaglandin G/H synthase 1	5742
Tenoxicam	Prostaglandin G/H synthase 2	5743
Tenoxicam	Prostaglandin G/H synthase 1	5742
Piroxicam	Prostaglandin G/H synthase 2	5743
Piroxicam	Prostaglandin G/H synthase 1	5742
Fenoprofen	Prostaglandin G/H synthase 1	5742
Fenoprofen	Prostaglandin G/H synthase 2	5743
Etodolac	Prostaglandin G/H synthase 2	5743
Etodolac	Prostaglandin G/H synthase 1	5742
Etodolac	Retinoic acid receptor RXR-alpha	6256
Mefenamic acid	Prostaglandin G/H synthase 2	5743
Mefenamic acid	Prostaglandin G/H synthase 1	5742
Naproxen	Prostaglandin G/H synthase 2	5743
Naproxen	Prostaglandin G/H synthase 1	5742
Sulfasalazine	Arachidonate 5-lipoxygenase	240
Sulfasalazine	Prostaglandin G/H synthase 2	5743
Sulfasalazine	Prostaglandin G/H synthase 1	5742
Sulfasalazine	Peroxisome proliferator-activated receptor gamma	5468
Sulfasalazine	Inhibitor of nuclear factor kappa-B kinase subunit alpha	1147
Sulfasalazine	Inhibitor of nuclear factor kappa-B kinase subunit beta	3551
Sulfasalazine	Cystine/glutamate transporter	23657
Sulfasalazine	"Acetyl-CoA acetyltransferase, mitochondrial"	6646
Sulfasalazine	"Acetyl-CoA acetyltransferase, mitochondrial"	4143
Sulfasalazine	Thromboxane-A synthase	6916
Sulfasalazine	Phospholipase A2	5319
Sulfasalazine	Phospholipase A2	8398
Phenylbutazone	Prostaglandin G/H synthase 2	5743
Phenylbutazone	Prostacyclin synthase	5740
Phenylbutazone	Prostaglandin G/H synthase 1	5742
Meclofenamic acid	Prostaglandin G/H synthase 1	5742
Meclofenamic acid	Prostaglandin G/H synthase 2	5743
Meclofenamic acid	Arachidonate 5-lipoxygenase	240
Meclofenamic acid	Potassium voltage-gated channel subfamily KQT member 2	3785
Meclofenamic acid	Potassium voltage-gated channel subfamily KQT member 3	3786
Methylprednisolone	Glucocorticoid receptor	2908
Methylprednisolone	Glucocorticoid receptor	23493
Oxaprozin	Prostaglandin G/H synthase 1	5742
Oxaprozin	Prostaglandin G/H synthase 2	5743
Azathioprine	Hypoxanthine-guanine phosphoribosyltransferase	3251
Leflunomide	"Dihydroorotate dehydrogenase (quinone), mitochondrial"	1723
Leflunomide	Aryl hydrocarbon receptor	196
Leflunomide	Protein-tyrosine kinase 2-beta	2185
Abatacept	T-lymphocyte activation antigen CD80	941
Abatacept	T-lymphocyte activation antigen CD86	942
Salsalate	Prostaglandin G/H synthase 2	5743
Salsalate	Prostaglandin G/H synthase 1	5742
Hydroxychloroquine	Toll-like receptor 7	51284

Hydroxychloroquine	Toll-like receptor 9	54106
Etoricoxib	Prostaglandin G/H synthase 2	5743
Temsirolimus	Serine/threonine-protein kinase mTOR	2475
Belatacept	T-lymphocyte activation antigen CD86	942
Belatacept	T-lymphocyte activation antigen CD80	941

Supplementary Table S5 Disease genes and supplied database. Genes associated with RA, announced as disease genes were collected from three databases: OMIM (http://www.omim.org), GAD (http://geneticassociationdb.nih.gov/) and KEGG (http://www.kegg.jp). This table lists the Gene ID of 675 disease genes and data source.

Gene ID	database	Gene ID	database	Gene ID	database	Gene ID	database
2	GAD	3303	GAD	6309	GAD	23275	GAD
10	GAD	3305	GAD	6311	GAD	23406	GAD
54	KEGG	3329	GAD	6347	KEGG,OMIM	23495	OMIM
100	GAD	3356	GAD	6348	GAD	23517	GAD
135	GAD	3383	KEGG,GAD	6349	KEGG,GAD	23526	GAD
142	GAD	3385	GAD	6352	KEGG,GAD	23534	GAD
154	GAD	3394	GAD	6364	KEGG	23545	KEGG
155	GAD	3456	GAD	6366	GAD	23569	GAD,OMIM
176	GAD	3458	KEGG,GAD,OMIM	6372	KEGG	23601	OMIM
199	GAD	3459	GAD	6374	KEGG	25898	GAD
214	OMIM	3478	OMIM	6387	KEGG,OMIM	26147	GAD
270	GAD	3514	OMIM	6399	OMIM	26191	GAD,OMIM
284	KEGG	3516	GAD	6401	GAD	26525	GAD
306	GAD	3551	GAD	6441	GAD	26762	GAD
326	GAD	3552	KEGG,GAD	6470	GAD	27164	GAD
331	GAD	3553	KEGG,GAD,OMIM	6504	OMIM	27177	GAD
348	GAD	3554	GAD	6538	GAD	27178	GAD
367	GAD	3557	GAD,OMIM	6556	GAD,OMIM	27179	GAD
471	GAD	3558	GAD	6573	GAD	28511	GAD
523	KEGG	3559	GAD	6583	GAD,OMIM	28512	GAD
525	KEGG	3560	GAD,OMIM	6584	GAD	28774	GAD
526	KEGG	3561	OMIM	6648	GAD	29113	GAD
527	KEGG	3562	GAD,OMIM	6649	GAD	29126	GAD
528	KEGG	3565	GAD	6696	GAD,OMIM	29800	GAD
529	KEGG	3566	GAD	6772	OMIM	29851	GAD
533	KEGG	3569	KEGG,GAD,OMIM	6774	OMIM	29943	OMIM
534	KEGG	3570	GAD	6775	GAD,OMIM	29945	GAD
535	KEGG	3572	GAD,OMIM	6778	OMIM	29953	GAD
537	KEGG	3575	GAD	6868	GAD,OMIM	30009	GAD,OMIM
558	OMIM	3576	KEGG,GAD	6885	GAD	30835	GAD
567	GAD	3577	GAD	6890	GAD	50617	KEGG
608	GAD	3579	GAD	6891	GAD	50619	OMIM
636	GAD	3581	GAD	6938	OMIM	50650	GAD
639	GAD	3586	GAD,OMIM	6957	GAD	50852	OMIM
640	GAD	3587	GAD	7010	KEGG	51135	GAD
650	OMIM	3589	KEGG	7018	GAD	51206	OMIM
654	OMIM	3592	GAD,OMIM	7026	GAD	51266	GAD
683	OMIM	3593	GAD	7036	GAD	51284	GAD
684	OMIM	3594	GAD	7040	KEGG,GAD	51311	GAD
840	GAD	3600	KEGG,GAD	7043	KEGG	51561	KEGG,GAD

847	GAD	3604	OMIM	7076	GAD	51606	KEGG
861	GAD	3605	KEGG,GAD,OMIM	7077	GAD	51702	OMIM
871	OMIM	3606	KEGG,GAD,OMIM	7079	GAD	51744	GAD,OMIM
901	GAD	3615	GAD	7096	GAD	53347	GAD
914	GAD	3630	GAD	7097	KEGG,GAD	53904	GAD
917	GAD	3654	GAD	7098	GAD	54106	GAD
919	GAD	3663	GAD,OMIM	7099	KEGG,GAD	54332	GAD
920	GAD	3683	KEGG	7100	GAD	54414	GAD,OMIM
929	GAD	3685	GAD	7103	GAD,OMIM	54469	GAD
930	GAD	3689	KEGG,OMIM	7124	KEGG,GAD,OMIM	54826	GAD
933	GAD	3704	GAD	7126	GAD	54829	GAD
934	GAD	3710	GAD	7128	GAD,OMIM	54899	GAD
940	KEGG,GAD	3725	KEGG	7130	OMIM	55024	GAD
941	KEGG,GAD	3798	GAD	7132	GAD,OMIM	55068	GAD
942	KEGG,GAD	3802	GAD	7133	GAD	55340	GAD
958	GAD	3803	GAD	7148	GAD,OMIM	55607	GAD
959	GAD	3804	GAD	7157	GAD,OMIM	55722	GAD
960	OMIM	3805	GAD	7185	GAD,OMIM	55801	GAD
965	GAD	3806	GAD	7188	GAD	55829	GAD
969	GAD	3807	GAD	7189	GAD	55937	GAD
1009	OMIM	3808	GAD	7220	OMIM	56005	OMIM
1012	GAD	3809	GAD	7224	OMIM	56244	GAD
1021	GAD	3810	GAD	7227	GAD	56300	GAD
1026	OMIM	3811	GAD	7274	GAD	56832	GAD
1030	GAD	3812	GAD	7291	OMIM	57140	GAD
1066	OMIM	3813	GAD	7292	GAD	57217	GAD
1108	OMIM	3821	GAD	7297	GAD	57579	GAD
1139	OMIM	3822	GAD	7298	GAD	57593	GAD
1147	GAD	3824	GAD	7317	GAD	57623	GAD
1231	GAD	3899	GAD,OMIM	7351	GAD	57650	OMIM
1232	GAD	3957	GAD	7356	GAD	57827	GAD
1233	GAD	3958	OMIM	7412	GAD	58189	GAD
1234	GAD,OMIM	3983	GAD	7421	GAD	59067	GAD
1235	GAD	4026	GAD	7422	KEGG,GAD,OMIM	59331	OMIM
1244	GAD	4041	GAD	7432	OMIM	60412	GAD
1280	GAD	4049	GAD	7454	OMIM	60468	GAD,OMIM
1298	GAD	4050	KEGG,GAD	7458	GAD	64127	GAD
1302	GAD	4118	GAD	7515	GAD	64135	GAD
1401	GAD	4146	OMIM	7535	OMIM	65217	GAD
1432	GAD	4153	GAD	7538	GAD,OMIM	79136	GAD
1435	KEGG	4193	GAD	7728	GAD	79155	GAD
1437	KEGG,GAD	4210	GAD,OMIM	7913	GAD,OMIM	79258	GAD

1460	GAD	4214	GAD	7916	GAD	79679	GAD
1470	GAD	4261	GAD,OMIM	7917	GAD	79722	GAD
1482	GAD	4267	GAD	7918	GAD	79728	GAD
1493	KEGG,GAD,OMIM	4276	GAD	7919	GAD	79817	GAD
1512	KEGG,GAD	4277	GAD	7920	GAD	79837	GAD
1514	KEGG	4282	GAD,OMIM	7940	OMIM	80018	GAD
1524	GAD	4312	KEGG,GAD	8518	GAD	80740	GAD
1544	GAD	4313	GAD	8543	GAD	80741	GAD
1557	GAD	4314	KEGG,GAD	8547	GAD	80863	GAD
1559	GAD	4316	GAD	8569	GAD	81545	GAD
1585	GAD	4318	GAD	8600	KEGG,GAD	83648	GAD
1586	GAD	4321	GAD	8651	GAD	84159	GAD
1588	GAD	4322	GAD	8718	OMIM	84162	GAD
1589	GAD	4323	OMIM	8737	GAD	84447	OMIM
1630	GAD	4350	GAD	8741	KEGG,GAD,OMIM	84516	GAD
1636	GAD	4363	GAD	8764	GAD	84619	GAD
1719	GAD	4439	GAD	8771	GAD	84639	GAD
1723	GAD	4522	GAD	8792	KEGG,GAD	84722	GAD
1777	GAD,OMIM	4524	GAD	8807	GAD	84868	GAD
1789	GAD	4540	GAD	8836	GAD	89978	GAD
1826	GAD	4543	GAD	8838	OMIM	90355	GAD
1843	GAD	4544	GAD	8887	GAD	90423	KEGG
1906	GAD	4548	GAD	8914	GAD	90865	OMIM
1968	GAD	4552	GAD	8927	GAD	91975	GAD
2065	GAD	4615	GAD	8986	GAD	112744	GAD
2099	GAD	4706	GAD	8992	KEGG	114548	GAD,OMIM
2100	GAD	4790	GAD	9020	GAD,OMIM	114609	GAD
2108	GAD	4791	GAD	9021	GAD,OMIM	115352	GAD,OMIM
2113	GAD	4792	GAD	9047	GAD	115653	GAD
2162	GAD	4793	GAD	9051	GAD	116154	GAD
2212	GAD	4794	GAD	9114	KEGG	117289	GAD
2213	GAD	4795	GAD,OMIM	9173	GAD,OMIM	122402	GAD
2214	GAD,OMIM	4808	GAD	9231	GAD	122618	GAD
2215	GAD,OMIM	4843	GAD	9235	OMIM	122773	GAD
2219	GAD	4846	GAD	9252	GAD	122953	GAD
2220	GAD	4855	GAD	9261	GAD,OMIM	124626	GAD
2244	GAD	4929	OMIM	9296	KEGG	126410	GAD
2302	GAD	4982	GAD	9315	GAD	127124	KEGG
2312	GAD	5027	GAD	9348	GAD	127795	GAD
2321	KEGG,OMIM	5054	GAD	9374	GAD	132612	GAD
2335	OMIM	5079	GAD	9378	GAD	135228	GAD
2348	GAD	5133	GAD	9398	GAD	140805	OMIM
2353	KEGG,OMIM	5138	GAD	9444	GAD	144811	OMIM
2356	GAD	5211	GAD	9536	OMIM	147372	GAD
2638	GAD	5241	GAD	9550	KEGG	148738	GAD

2643	GAD	5243	GAD	9641	GAD	149233	GAD
2693	GAD	5265	GAD	9683	GAD	151888	GAD
2784	GAD	5294	OMIM	9692	GAD	155066	KEGG
2804	OMIM	5320	OMIM	9899	GAD	161357	GAD
2814	OMIM	5321	OMIM	9935	GAD	163351	GAD
2821	OMIM	5327	GAD	10010	GAD	167826	GAD
2833	GAD	5328	GAD	10019	GAD	200734	GAD
2908	GAD	5444	GAD	10148	OMIM	201294	GAD
2919	KEGG	5453	GAD	10203	GAD	203228	GAD
2944	GAD	5468	GAD	10266	GAD	221687	GAD
2948	GAD	5551	GAD	10312	KEGG	245972	KEGG
2950	GAD	5579	GAD	10333	GAD	245973	KEGG
2952	GAD	5583	GAD	10344	GAD	259308	GAD
2990	OMIM	5588	GAD,OMIM	10347	GAD	282890	GAD
3002	GAD	5603	GAD	10454	GAD	283635	GAD
3039	GAD	5606	GAD	10461	OMIM	342132	GAD
3075	GAD	5608	GAD	10464	GAD	345611	GAD
3077	GAD	5617	GAD	10512	OMIM	353143	GAD
3106	GAD	5654	GAD,OMIM	10599	GAD	353144	GAD
3107	GAD,OMIM	5685	GAD	10640	GAD	374378	GAD
3108	KEGG,GAD	5687	GAD	10665	GAD	387082	GAD
3109	KEGG,GAD	5696	OMIM	10666	GAD	387129	GAD
3111	KEGG,GAD	5698	GAD	10673	KEGG,GAD,OMIM	389203	GAD
3112	KEGG	5734	GAD	10678	GAD	391370	GAD
3113	KEGG	5743	GAD	10746	OMIM	399716	GAD
3115	KEGG,GAD	5771	GAD	10859	GAD,OMIM	406938	GAD
3117	KEGG,GAD,OMIM	5777	GAD	10956	GAD	414062	KEGG
3118	KEGG,GAD	5788	GAD	10986	OMIM	643365	GAD
3119	KEGG,GAD	5873	GAD	11061	GAD	653361	OMIM
3120	GAD	5891	GAD	11122	GAD	728411	GAD
3122	KEGG,GAD	5896	GAD	11167	GAD,OMIM	729230	OMIM
3123	KEGG,GAD,OMIM	5919	GAD	11170	GAD	100130458	GAD
3124	GAD	5966	GAD,OMIM	11213	GAD	100132285	GAD,OMIM
3125	KEGG	5970	GAD	11240	OMIM	100188846	OMIM
3126	KEGG	5971	GAD	11270	GAD	100196918	OMIM
3127	KEGG,GAD	5981	GAD	11278	GAD	100306940	OMIM
3135	GAD,OMIM	5996	GAD	22900	GAD	100507436	GAD
3158	GAD	6014	GAD	22914	GAD		
3162	GAD	6147	OMIM	22943	OMIM		
3251	GAD	6176	GAD	23118	GAD		
3267	GAD	6280	OMIM	23228	GAD		
3273	OMIM	6288	GAD,OMIM	23274	GAD		

Supplement Table S6 The overlapped 7 targets of GSZ formula, RA-related disease genes and drug targets. 7 targets were found to be in common in comparasion between the 1,327 GSZ formula targets with the 79 targets of FDA 30 approved RA drugs.

**target name**	**abbreviation**
conserved helix-loop-helix ubiquitous kinase	CHUK
dihydroorotate dehydrogenase	DHODH
nuclear receptor subfamily 3, group C, member 1	NR3C1
peroxisome proliferator-activated recepto	PPARG
prostaglandin-endoperoxide synthase 2	PTGS2
tumor necrosis factor	TNF
phospholipase A2, group IIA	PLA2G2A

Supplementary Table S7 Genes directly interacted with p50. In the network generated by Cytoscape, p50, defined as both RA disease gene and GSZ target, directly interacted with 7 genes.

**target name**	**abbreviation**
signal transducer and activator of transcription 3	HIES
nuclear factor of kappa light polypeptide gene enhancer in B-cells inhibitor, alpha	NFKBI
ubiquitin-conjugating enzyme E2D 3	UBCH5C
poly (ADP-ribose) polymerase 1	pADPRT-1
protein phosphatase 4, catalytic subunit	PPX
E74-like factor 1 (ets domain transcription factor)	ELF1
receptor (TNFRSF)-interacting serine-threonine kinase 1	RIP1

Supplementary Table S8 Disease genes interacted with either MLRW or HLA-DRB1. In the network generated by Cytoscape, two formula targets MLRW and HLA-DRB1 directly or indirectly interact with five disease genes.

**target name**	**abbreviation**
major histocompatibility complex, class II, DR beta 5	HLA-DRB5
major histocompatibility complex, class II, DM beta	HLA-DMB, RING7
major histocompatibility complex, class II, DM alpha	HLA-DMA, RING6
major histocompatibility complex, class II, DP alpha 1	HLA-DPA1, PLT1
major histocompatibility complex, class II, DP beta 1	HLA-DPB

Supplement Table S9 7 disease genes directly interact with MSF. In the network generated by Cytoscape, MSF directly interacted with 7 disease genes.

**target name**	**abbreviation**
matrix metallopeptidase 3	STR1
caspase recruitment domain family, member 8	TUCAN
chemokine (C-X-C motif) ligand 12	TPAR1
HtrA serine peptidase 1	PRSS11
integrin, alpha V	VTNR
matrix metallopeptidase 2	TBE-1
phospholipase A2, group IIA	sPLA2

Supplementary Table S10 Clinical information of 10 RA patients. Gender, stage of disease, serum levels of RF, ESR and CRP, and some other parameters of every patient were recorded and listed in the table.

**patient**	**SEX**	**AGE (y)**	**WEIGHT (kg)**	**HIGH (m)**	**BMI**	**Stage**	**RF T0 (IU/ml)**	**RF T1 (IU/ml)**	**RF T2 (IU/ml)**	**RF T3 (IU/ml)**	**CRP T0 (mg/L)**	**CRP T1 (mg/L)**	**CRP T2 (mg/L)**	**CRP T3 (mg/L)**	**ESR T0 (mm/h)**	**ESR T1 (mm/h)**	**ESR T2 (mm/h)**	**ESR T3 (mm/h)**
P1	F	63	62	1.65	22.8	II	233.5	17	-	-	4.3	4.8	-	-	15	4.8	-	-
P2	F	46	56	1.66	20.3	II	82.5	131.8	112.5	48.5	1	1.4	0.9	1.7	28	23	29	24
P3	M	65	60	1.75	19.6	II	207	30	25.7	21.3	33	-	-	-	43	43	-	-
P4	M	59	61	1.72	20.6	II	31.5	53.2	-	-	27	42.73	-	-	18	30	-	-
P5	F	53	56	1.62	21.3	III	65	19.3	-	-	2.4	1.9	-	-	46	90	-	-
P6	F	67	53	1.6	20.7	II	443	285	-	-	-	-	-	-	68	-	-	-
P7	F	71	63	1.68	22.3	II	541	82.6	23.8	-	-	40	5	-	96	17	19	-
P8	F	29	50	1.62	19.1	II	71.8	63.7	-	-	3	1	-	-	7	6	-	-
P9	F	62	47	1.57	19.1	II	311	166.1	-	-	2.3	3	-	-	13	-	-	-
P10	F	52	52.5	1.67	18.8	I	74.9		-	-	-	3.26	-	-	35	26	-	-

Supplement Table 11 1080 microarrays collected from GEO database. 1080 microarrays were collected from GEO database and then classified as 174 normal samples (WT), 312 RA disease samples (RA_ONLY), 551 RA treatment samples (RA_TREAT) and 42 other RA related samples (RA_OTHER).

GSM ID	Group	GSE ID	GPL ID	GSM ID	Group	GSE ID	GPL ID
GSM253285	WT	GSE10024	GPL96	GSM1320250	RA_TREAT	GSE54629	GPL6244
GSM253286	WT	GSE10024	GPL96	GSM1320251	RA_TREAT	GSE54629	GPL6244
GSM265436	WT	GSE10500	GPL8300	GSM1320252	RA_TREAT	GSE54629	GPL6244
GSM265439	WT	GSE10500	GPL8300	GSM1320253	RA_TREAT	GSE54629	GPL6244
GSM265445	WT	GSE10500	GPL8300	GSM1320254	RA_TREAT	GSE54629	GPL6244
GSM302859	WT	GSE12021	GPL96	GSM1320255	RA_TREAT	GSE54629	GPL6244
GSM302864	WT	GSE12021	GPL96	GSM1320256	RA_TREAT	GSE54629	GPL6244
GSM302866	WT	GSE12021	GPL96	GSM1320257	RA_TREAT	GSE54629	GPL6244
GSM302870	WT	GSE12021	GPL96	GSM1320258	RA_TREAT	GSE54629	GPL6244
GSM303522	WT	GSE12021	GPL96	GSM1320259	RA_TREAT	GSE54629	GPL6244
GSM303523	WT	GSE12021	GPL96	GSM1320260	RA_TREAT	GSE54629	GPL6244
GSM303525	WT	GSE12021	GPL96	GSM1320261	RA_TREAT	GSE54629	GPL6244
GSM303531	WT	GSE12021	GPL96	GSM1320262	RA_TREAT	GSE54629	GPL6244
GSM303533	WT	GSE12021	GPL96	GSM1320263	RA_TREAT	GSE54629	GPL6244
GSM389705	WT	GSE15573	GPL6102	GSM1320264	RA_TREAT	GSE54629	GPL6244
GSM389707	WT	GSE15573	GPL6102	GSM1320265	RA_TREAT	GSE54629	GPL6244
GSM389709	WT	GSE15573	GPL6102	GSM1320266	RA_TREAT	GSE54629	GPL6244
GSM389710	WT	GSE15573	GPL6102	GSM1320267	RA_TREAT	GSE54629	GPL6244
GSM389712	WT	GSE15573	GPL6102	GSM1320268	RA_TREAT	GSE54629	GPL6244
GSM389713	WT	GSE15573	GPL6102	GSM1320269	RA_TREAT	GSE54629	GPL6244
GSM389715	WT	GSE15573	GPL6102	GSM1320270	RA_TREAT	GSE54629	GPL6244
GSM389718	WT	GSE15573	GPL6102	GSM1320271	RA_TREAT	GSE54629	GPL6244
GSM389720	WT	GSE15573	GPL6102	GSM1320272	RA_TREAT	GSE54629	GPL6244
GSM389723	WT	GSE15573	GPL6102	GSM1320273	RA_TREAT	GSE54629	GPL6244
GSM389725	WT	GSE15573	GPL6102	GSM1320274	RA_TREAT	GSE54629	GPL6244
GSM389728	WT	GSE15573	GPL6102	GSM1320275	RA_TREAT	GSE54629	GPL6244
GSM389729	WT	GSE15573	GPL6102	GSM1320276	RA_TREAT	GSE54629	GPL6244
GSM389732	WT	GSE15573	GPL6102	GSM1320277	RA_TREAT	GSE54629	GPL6244
GSM389734	WT	GSE15573	GPL6102	GSM1320278	RA_TREAT	GSE54629	GPL6244
GSM34379	WT	GSE1919	GPL91	GSM1320279	RA_TREAT	GSE54629	GPL6244
GSM34383	WT	GSE1919	GPL91	GSM1320280	RA_TREAT	GSE54629	GPL6244
GSM34385	WT	GSE1919	GPL91	GSM1320281	RA_TREAT	GSE54629	GPL6244
GSM34388	WT	GSE1919	GPL91	GSM1320282	RA_TREAT	GSE54629	GPL6244
GSM34391	WT	GSE1919	GPL91	GSM1320283	RA_TREAT	GSE54629	GPL6244
GSM546086	WT	GSE21959	GPL4133	GSM1320284	RA_TREAT	GSE54629	GPL6244
GSM546087	WT	GSE21959	GPL4133	GSM1320285	RA_TREAT	GSE54629	GPL6244
GSM546088	WT	GSE21959	GPL4133	GSM1320286	RA_TREAT	GSE54629	GPL6244
GSM546089	WT	GSE21959	GPL4133	GSM1320287	RA_TREAT	GSE54629	GPL6244
GSM546090	WT	GSE21959	GPL4133	GSM1320288	RA_TREAT	GSE54629	GPL6244
GSM546091	WT	GSE21959	GPL4133	GSM1320289	RA_TREAT	GSE54629	GPL6244
GSM546098	WT	GSE21959	GPL4133	GSM1332221	RA_TREAT	GSE55235	GPL96
GSM546099	WT	GSE21959	GPL4133	GSM1332222	RA_TREAT	GSE55235	GPL96
GSM546100	WT	GSE21959	GPL4133	GSM1332223	RA_TREAT	GSE55235	GPL96
GSM546101	WT	GSE21959	GPL4133	GSM1332224	RA_TREAT	GSE55235	GPL96

GSM546102	WT	GSE21959	GPL4133	GSM1332225	RA_TREAT	GSE55235	GPL96
GSM546103	WT	GSE21959	GPL4133	GSM1332226	RA_TREAT	GSE55235	GPL96
GSM546104	WT	GSE21959	GPL4133	GSM1332227	RA_TREAT	GSE55235	GPL96
GSM546105	WT	GSE21959	GPL4133	GSM1332228	RA_TREAT	GSE55235	GPL96
GSM546118	WT	GSE21959	GPL4133	GSM1332229	RA_TREAT	GSE55235	GPL96
GSM546119	WT	GSE21959	GPL4133	GSM1332230	RA_TREAT	GSE55235	GPL96
GSM546120	WT	GSE21959	GPL4133	GSM1337314	RA_TREAT	GSE55457	GPL96
GSM546121	WT	GSE21959	GPL4133	GSM1337315	RA_TREAT	GSE55457	GPL96
GSM677348	WT	GSE27390	GPL570	GSM1337316	RA_TREAT	GSE55457	GPL96
GSM677349	WT	GSE27390	GPL570	GSM1337317	RA_TREAT	GSE55457	GPL96
GSM677350	WT	GSE27390	GPL570	GSM1337318	RA_TREAT	GSE55457	GPL96
GSM677351	WT	GSE27390	GPL570	GSM1337319	RA_TREAT	GSE55457	GPL96
GSM677352	WT	GSE27390	GPL570	GSM1337320	RA_TREAT	GSE55457	GPL96
GSM677353	WT	GSE27390	GPL570	GSM1337321	RA_TREAT	GSE55457	GPL96
GSM677354	WT	GSE27390	GPL570	GSM1337322	RA_TREAT	GSE55457	GPL96
GSM677355	WT	GSE27390	GPL570	GSM1337323	RA_TREAT	GSE55457	GPL96
GSM677356	WT	GSE27390	GPL570	GSM1337324	RA_TREAT	GSE55457	GPL96
GSM677357	WT	GSE27390	GPL570	GSM1337325	RA_TREAT	GSE55457	GPL96
GSM737470	WT	GSE29746	GPL4133	GSM1337326	RA_TREAT	GSE55457	GPL96
GSM737471	WT	GSE29746	GPL4133	GSM1339618	RA_TREAT	GSE55584	GPL96
GSM737472	WT	GSE29746	GPL4133	GSM1339619	RA_TREAT	GSE55584	GPL96
GSM737473	WT	GSE29746	GPL4133	GSM1339620	RA_TREAT	GSE55584	GPL96
GSM737474	WT	GSE29746	GPL4133	GSM1339621	RA_TREAT	GSE55584	GPL96
GSM737475	WT	GSE29746	GPL4133	GSM1339622	RA_TREAT	GSE55584	GPL96
GSM737476	WT	GSE29746	GPL4133	GSM1339623	RA_TREAT	GSE55584	GPL96
GSM737477	WT	GSE29746	GPL4133	GSM1339624	RA_TREAT	GSE55584	GPL96
GSM737478	WT	GSE29746	GPL4133	GSM1339625	RA_TREAT	GSE55584	GPL96
GSM737479	WT	GSE29746	GPL4133	GSM1339626	RA_TREAT	GSE55584	GPL96
GSM737480	WT	GSE29746	GPL4133	GSM1339627	RA_TREAT	GSE55584	GPL96
GSM1100843	WT	GSE45291	GPL13158	GSM1381430	RA_TREAT	GSE57386	GPL13158
GSM1100844	WT	GSE45291	GPL13158	GSM1381431	RA_TREAT	GSE57386	GPL13158
GSM1100845	WT	GSE45291	GPL13158	GSM1381432	RA_TREAT	GSE57386	GPL13158
GSM1100846	WT	GSE45291	GPL13158	GSM1381433	RA_TREAT	GSE57386	GPL13158
GSM1100847	WT	GSE45291	GPL13158	GSM1381610	RA_TREAT	GSE57383	GPL13158
GSM1100848	WT	GSE45291	GPL13158	GSM1381611	RA_TREAT	GSE57383	GPL13158
GSM1100849	WT	GSE45291	GPL13158	GSM1381612	RA_TREAT	GSE57383	GPL13158
GSM1100850	WT	GSE45291	GPL13158	GSM1381613	RA_TREAT	GSE57383	GPL13158
GSM1100851	WT	GSE45291	GPL13158	GSM1381614	RA_TREAT	GSE57383	GPL13158
GSM1100852	WT	GSE45291	GPL13158	GSM1381615	RA_TREAT	GSE57383	GPL13158
GSM1100853	WT	GSE45291	GPL13158	GSM1381617	RA_TREAT	GSE57383	GPL13158
GSM1100854	WT	GSE45291	GPL13158	GSM1381618	RA_TREAT	GSE57383	GPL13158
GSM1100855	WT	GSE45291	GPL13158	GSM1381619	RA_TREAT	GSE57383	GPL13158
GSM1100856	WT	GSE45291	GPL13158	GSM1381622	RA_TREAT	GSE57383	GPL13158
GSM1100857	WT	GSE45291	GPL13158	GSM1381623	RA_TREAT	GSE57383	GPL13158
GSM1100858	WT	GSE45291	GPL13158	GSM1381625	RA_TREAT	GSE57383	GPL13158
GSM1100859	WT	GSE45291	GPL13158	GSM1381626	RA_TREAT	GSE57383	GPL13158
GSM1100860	WT	GSE45291	GPL13158	GSM1381627	RA_TREAT	GSE57383	GPL13158

GSM1100861	WT	GSE45291	GPL13158	GSM1381628	RA_TREAT	GSE57383	GPL13158
GSM1100862	WT	GSE45291	GPL13158	GSM1381630	RA_TREAT	GSE57383	GPL13158
GSM101869	WT	GSE4588	GPL570	GSM1381631	RA_TREAT	GSE57383	GPL13158
GSM101870	WT	GSE4588	GPL570	GSM1381633	RA_TREAT	GSE57383	GPL13158
GSM101871	WT	GSE4588	GPL570	GSM1382133	RA_TREAT	GSE57405	GPL13158
GSM101872	WT	GSE4588	GPL570	GSM1382134	RA_TREAT	GSE57405	GPL13158
GSM101873	WT	GSE4588	GPL570	GSM1382135	RA_TREAT	GSE57405	GPL13158
GSM101874	WT	GSE4588	GPL570	GSM1382136	RA_TREAT	GSE57405	GPL13158
GSM101875	WT	GSE4588	GPL570	GSM1382137	RA_TREAT	GSE57405	GPL13158
GSM101876	WT	GSE4588	GPL570	GSM1382138	RA_TREAT	GSE57405	GPL13158
GSM101877	WT	GSE4588	GPL570	GSM1382139	RA_TREAT	GSE57405	GPL13158
GSM101878	WT	GSE4588	GPL570	GSM1382140	RA_TREAT	GSE57405	GPL13158
GSM101962	WT	GSE4588	GPL570	GSM1382141	RA_TREAT	GSE57405	GPL13158
GSM101963	WT	GSE4588	GPL570	GSM1382142	RA_TREAT	GSE57405	GPL13158
GSM101964	WT	GSE4588	GPL570	GSM1382143	RA_TREAT	GSE57405	GPL13158
GSM101965	WT	GSE4588	GPL570	GSM1382144	RA_TREAT	GSE57405	GPL13158
GSM101966	WT	GSE4588	GPL570	GSM1382145	RA_TREAT	GSE57405	GPL13158
GSM101967	WT	GSE4588	GPL570	GSM1382146	RA_TREAT	GSE57405	GPL13158
GSM101968	WT	GSE4588	GPL570	GSM1382147	RA_TREAT	GSE57405	GPL13158
GSM101969	WT	GSE4588	GPL570	GSM1382148	RA_TREAT	GSE57405	GPL13158
GSM101970	WT	GSE4588	GPL570	GSM1382149	RA_TREAT	GSE57405	GPL13158
GSM1202491	WT	GSE49604	GPL8432	GSM1382150	RA_TREAT	GSE57405	GPL13158
GSM1202492	WT	GSE49604	GPL8432	GSM1419699	RA_TREAT	GSE58795	GPL10379
GSM1332201	WT	GSE55235	GPL96	GSM1419700	RA_TREAT	GSE58795	GPL10379
GSM1332202	WT	GSE55235	GPL96	GSM1419701	RA_TREAT	GSE58795	GPL10379
GSM1332203	WT	GSE55235	GPL96	GSM1419702	RA_TREAT	GSE58795	GPL10379
GSM1332204	WT	GSE55235	GPL96	GSM1419703	RA_TREAT	GSE58795	GPL10379
GSM1332205	WT	GSE55235	GPL96	GSM1419704	RA_TREAT	GSE58795	GPL10379
GSM1332206	WT	GSE55235	GPL96	GSM1419705	RA_TREAT	GSE58795	GPL10379
GSM1332207	WT	GSE55235	GPL96	GSM1419706	RA_TREAT	GSE58795	GPL10379
GSM1332208	WT	GSE55235	GPL96	GSM1419707	RA_TREAT	GSE58795	GPL10379
GSM1332209	WT	GSE55235	GPL96	GSM1419708	RA_TREAT	GSE58795	GPL10379
GSM1332210	WT	GSE55235	GPL96	GSM1419709	RA_TREAT	GSE58795	GPL10379
GSM1337304	WT	GSE55457	GPL96	GSM1419710	RA_TREAT	GSE58795	GPL10379
GSM1337305	WT	GSE55457	GPL96	GSM1419711	RA_TREAT	GSE58795	GPL10379
GSM1337306	WT	GSE55457	GPL96	GSM1419712	RA_TREAT	GSE58795	GPL10379
GSM1337307	WT	GSE55457	GPL96	GSM1419713	RA_TREAT	GSE58795	GPL10379
GSM1337308	WT	GSE55457	GPL96	GSM1419714	RA_TREAT	GSE58795	GPL10379
GSM1337309	WT	GSE55457	GPL96	GSM1419715	RA_TREAT	GSE58795	GPL10379
GSM1337310	WT	GSE55457	GPL96	GSM1419716	RA_TREAT	GSE58795	GPL10379
GSM1337311	WT	GSE55457	GPL96	GSM1419717	RA_TREAT	GSE58795	GPL10379
GSM1337312	WT	GSE55457	GPL96	GSM1419718	RA_TREAT	GSE58795	GPL10379
GSM1337313	WT	GSE55457	GPL96	GSM1419719	RA_TREAT	GSE58795	GPL10379
GSM1381524	WT	GSE57383	GPL13158	GSM1419720	RA_TREAT	GSE58795	GPL10379
GSM1381525	WT	GSE57383	GPL13158	GSM1419721	RA_TREAT	GSE58795	GPL10379
GSM1381526	WT	GSE57383	GPL13158	GSM1419722	RA_TREAT	GSE58795	GPL10379
GSM1381527	WT	GSE57383	GPL13158	GSM1419723	RA_TREAT	GSE58795	GPL10379

GSM1381528	WT	GSE57383	GPL13158	GSM1419724	RA_TREAT	GSE58795	GPL10379
GSM1381529	WT	GSE57383	GPL13158	GSM1419725	RA_TREAT	GSE58795	GPL10379
GSM1381530	WT	GSE57383	GPL13158	GSM1419726	RA_TREAT	GSE58795	GPL10379
GSM1381531	WT	GSE57383	GPL13158	GSM1419727	RA_TREAT	GSE58795	GPL10379
GSM1381532	WT	GSE57383	GPL13158	GSM1419728	RA_TREAT	GSE58795	GPL10379
GSM1381533	WT	GSE57383	GPL13158	GSM1419729	RA_TREAT	GSE58795	GPL10379
GSM1381534	WT	GSE57383	GPL13158	GSM1419730	RA_TREAT	GSE58795	GPL10379
GSM1381535	WT	GSE57383	GPL13158	GSM1419731	RA_TREAT	GSE58795	GPL10379
GSM1381536	WT	GSE57383	GPL13158	GSM1419732	RA_TREAT	GSE58795	GPL10379
GSM1381537	WT	GSE57383	GPL13158	GSM1419733	RA_TREAT	GSE58795	GPL10379
GSM1381538	WT	GSE57383	GPL13158	GSM1419734	RA_TREAT	GSE58795	GPL10379
GSM1381539	WT	GSE57383	GPL13158	GSM1419735	RA_TREAT	GSE58795	GPL10379
GSM1381540	WT	GSE57383	GPL13158	GSM1419736	RA_TREAT	GSE58795	GPL10379
GSM1381541	WT	GSE57383	GPL13158	GSM1419737	RA_TREAT	GSE58795	GPL10379
GSM1381542	WT	GSE57383	GPL13158	GSM1419738	RA_TREAT	GSE58795	GPL10379
GSM1382105	WT	GSE57405	GPL13158	GSM1419739	RA_TREAT	GSE58795	GPL10379
GSM1382106	WT	GSE57405	GPL13158	GSM1419740	RA_TREAT	GSE58795	GPL10379
GSM1382107	WT	GSE57405	GPL13158	GSM1419741	RA_TREAT	GSE58795	GPL10379
GSM1382108	WT	GSE57405	GPL13158	GSM1419742	RA_TREAT	GSE58795	GPL10379
GSM1382109	WT	GSE57405	GPL13158	GSM1419743	RA_TREAT	GSE58795	GPL10379
GSM1382110	WT	GSE57405	GPL13158	GSM1419744	RA_TREAT	GSE58795	GPL10379
GSM1382111	WT	GSE57405	GPL13158	GSM1419745	RA_TREAT	GSE58795	GPL10379
GSM1382112	WT	GSE57405	GPL13158	GSM1419746	RA_TREAT	GSE58795	GPL10379
GSM1382113	WT	GSE57405	GPL13158	GSM1419747	RA_TREAT	GSE58795	GPL10379
GSM1382114	WT	GSE57405	GPL13158	GSM1419748	RA_TREAT	GSE58795	GPL10379
GSM1382115	WT	GSE57405	GPL13158	GSM1419749	RA_TREAT	GSE58795	GPL10379
GSM1382116	WT	GSE57405	GPL13158	GSM1419750	RA_TREAT	GSE58795	GPL10379
GSM1382117	WT	GSE57405	GPL13158	GSM1419751	RA_TREAT	GSE58795	GPL10379
GSM1382118	WT	GSE57405	GPL13158	GSM1419752	RA_TREAT	GSE58795	GPL10379
GSM1382119	WT	GSE57405	GPL13158	GSM1419753	RA_TREAT	GSE58795	GPL10379
GSM1382120	WT	GSE57405	GPL13158	GSM1419754	RA_TREAT	GSE58795	GPL10379
GSM1382121	WT	GSE57405	GPL13158	GSM1419755	RA_TREAT	GSE58795	GPL10379
GSM1382122	WT	GSE57405	GPL13158	GSM1419756	RA_TREAT	GSE58795	GPL10379
GSM1382123	WT	GSE57405	GPL13158	GSM1419757	RA_TREAT	GSE58795	GPL10379
GSM119289	WT	GSE7524	GPL96	GSM119293	RA_TREAT	GSE7524	GPL96
GSM119290	WT	GSE7524	GPL96	GSM119294	RA_TREAT	GSE7524	GPL96
GSM265020	RA_TREAT	GSE10500	GPL8300	GSM185526	RA_TREAT	GSE7669	GPL8300
GSM265036	RA_TREAT	GSE10500	GPL8300	GSM185527	RA_TREAT	GSE7669	GPL8300
GSM265361	RA_TREAT	GSE10500	GPL8300	GSM185528	RA_TREAT	GSE7669	GPL8300
GSM265363	RA_TREAT	GSE10500	GPL8300	GSM185529	RA_TREAT	GSE7669	GPL8300
GSM265366	RA_TREAT	GSE10500	GPL8300	GSM185530	RA_TREAT	GSE7669	GPL8300
GSM302872	RA_TREAT	GSE12021	GPL96	GSM11947	RA_TREAT	GSE766	GPL8300
GSM302882	RA_TREAT	GSE12021	GPL96	GSM11954	RA_TREAT	GSE766	GPL8300
GSM302896	RA_TREAT	GSE12021	GPL96	GSM11955	RA_TREAT	GSE766	GPL8300
GSM302900	RA_TREAT	GSE12021	GPL96	GSM11956	RA_TREAT	GSE766	GPL8300
GSM302924	RA_TREAT	GSE12021	GPL96	GSM11957	RA_TREAT	GSE766	GPL8300
GSM302927	RA_TREAT	GSE12021	GPL96	GSM11958	RA_TREAT	GSE766	GPL8300

GSM302933	RA_TREAT	GSE12021	GPL96	GSM253283	RA_OTHER	GSE10024	GPL96
GSM302943	RA_TREAT	GSE12021	GPL96	GSM253284	RA_OTHER	GSE10024	GPL96
GSM303364	RA_TREAT	GSE12021	GPL96	GSM566729	RA_OTHER	GSE22956	GPL6947
GSM303366	RA_TREAT	GSE12021	GPL96	GSM566730	RA_OTHER	GSE22956	GPL6947
GSM303368	RA_TREAT	GSE12021	GPL96	GSM566732	RA_OTHER	GSE22956	GPL6947
GSM303520	RA_TREAT	GSE12021	GPL96	GSM566733	RA_OTHER	GSE22956	GPL6947
GSM322613	RA_TREAT	GSE12860	GPL96	GSM566735	RA_OTHER	GSE22956	GPL6947
GSM322614	RA_TREAT	GSE12860	GPL96	GSM566736	RA_OTHER	GSE22956	GPL6947
GSM322615	RA_TREAT	GSE12860	GPL96	GSM566738	RA_OTHER	GSE22956	GPL6947
GSM322616	RA_TREAT	GSE12860	GPL96	GSM566739	RA_OTHER	GSE22956	GPL6947
GSM322617	RA_TREAT	GSE12860	GPL96	GSM786584	RA_OTHER	GSE31685	GPL6244
GSM322618	RA_TREAT	GSE12860	GPL96	GSM786585	RA_OTHER	GSE31685	GPL6244
GSM322619	RA_TREAT	GSE12860	GPL96	GSM786586	RA_OTHER	GSE31685	GPL6244
GSM322620	RA_TREAT	GSE12860	GPL96	GSM786587	RA_OTHER	GSE31685	GPL6244
GSM322621	RA_TREAT	GSE12860	GPL96	GSM786588	RA_OTHER	GSE31685	GPL6244
GSM322622	RA_TREAT	GSE12860	GPL96	GSM786589	RA_OTHER	GSE31685	GPL6244
GSM322623	RA_TREAT	GSE12860	GPL96	GSM1111504	RA_OTHER	GSE45665	GPL570
GSM322624	RA_TREAT	GSE12860	GPL96	GSM1111505	RA_OTHER	GSE45665	GPL570
GSM322625	RA_TREAT	GSE12860	GPL96	GSM1111506	RA_OTHER	GSE45665	GPL570
GSM322626	RA_TREAT	GSE12860	GPL96	GSM1111507	RA_OTHER	GSE45665	GPL570
GSM322627	RA_TREAT	GSE12860	GPL96	GSM1111508	RA_OTHER	GSE45665	GPL570
GSM322628	RA_TREAT	GSE12860	GPL96	GSM1111509	RA_OTHER	GSE45665	GPL570
GSM347841	RA_TREAT	GSE13837	GPL570	GSM1111510	RA_OTHER	GSE45665	GPL570
GSM347842	RA_TREAT	GSE13837	GPL570	GSM1111511	RA_OTHER	GSE45665	GPL570
GSM347843	RA_TREAT	GSE13837	GPL570	GSM101879	RA_OTHER	GSE4588	GPL570
GSM347844	RA_TREAT	GSE13837	GPL570	GSM101880	RA_OTHER	GSE4588	GPL570
GSM348017	RA_TREAT	GSE13837	GPL570	GSM101881	RA_OTHER	GSE4588	GPL570
GSM348018	RA_TREAT	GSE13837	GPL570	GSM101882	RA_OTHER	GSE4588	GPL570
GSM348020	RA_TREAT	GSE13837	GPL570	GSM101883	RA_OTHER	GSE4588	GPL570
GSM348022	RA_TREAT	GSE13837	GPL570	GSM101884	RA_OTHER	GSE4588	GPL570
GSM348024	RA_TREAT	GSE13837	GPL570	GSM101885	RA_OTHER	GSE4588	GPL570
GSM348027	RA_TREAT	GSE13837	GPL570	GSM101886	RA_OTHER	GSE4588	GPL570
GSM348028	RA_TREAT	GSE13837	GPL570	GSM102703	RA_OTHER	GSE4588	GPL570
GSM348029	RA_TREAT	GSE13837	GPL570	GSM102704	RA_OTHER	GSE4588	GPL570
GSM348031	RA_TREAT	GSE13837	GPL570	GSM102705	RA_OTHER	GSE4588	GPL570
GSM348034	RA_TREAT	GSE13837	GPL570	GSM102706	RA_OTHER	GSE4588	GPL570
GSM348035	RA_TREAT	GSE13837	GPL570	GSM102707	RA_OTHER	GSE4588	GPL570
GSM348036	RA_TREAT	GSE13837	GPL570	GSM102708	RA_OTHER	GSE4588	GPL570
GSM381194	RA_TREAT	GSE15258	GPL570	GSM102709	RA_OTHER	GSE4588	GPL570
GSM381195	RA_TREAT	GSE15258	GPL570	GSM1562203	RA_OTHER	GSE63995	GPL570
GSM381196	RA_TREAT	GSE15258	GPL570	GSM1562205	RA_OTHER	GSE63995	GPL570
GSM381197	RA_TREAT	GSE15258	GPL570	GSM1562207	RA_OTHER	GSE63995	GPL570
GSM381198	RA_TREAT	GSE15258	GPL570	GSM1562209	RA_OTHER	GSE63995	GPL570
GSM381199	RA_TREAT	GSE15258	GPL570	GSM253287	RA_ONLY	GSE10024	GPL96
GSM381200	RA_TREAT	GSE15258	GPL570	GSM253288	RA_ONLY	GSE10024	GPL96
GSM381201	RA_TREAT	GSE15258	GPL570	GSM322609	RA_ONLY	GSE12860	GPL96
GSM381202	RA_TREAT	GSE15258	GPL570	GSM322610	RA_ONLY	GSE12860	GPL96

GSM381203	RA_TREAT	GSE15258	GPL570	GSM322611	RA_ONLY	GSE12860	GPL96
GSM381204	RA_TREAT	GSE15258	GPL570	GSM322612	RA_ONLY	GSE12860	GPL96
GSM381205	RA_TREAT	GSE15258	GPL570	GSM347845	RA_ONLY	GSE13837	GPL570
GSM381206	RA_TREAT	GSE15258	GPL570	GSM348023	RA_ONLY	GSE13837	GPL570
GSM381207	RA_TREAT	GSE15258	GPL570	GSM348030	RA_ONLY	GSE13837	GPL570
GSM381208	RA_TREAT	GSE15258	GPL570	GSM348037	RA_ONLY	GSE13837	GPL570
GSM381209	RA_TREAT	GSE15258	GPL570	GSM350574	RA_ONLY	GSE13917	GPL570
GSM381210	RA_TREAT	GSE15258	GPL570	GSM350575	RA_ONLY	GSE13917	GPL570
GSM381211	RA_TREAT	GSE15258	GPL570	GSM350601	RA_ONLY	GSE13917	GPL570
GSM381212	RA_TREAT	GSE15258	GPL570	GSM350602	RA_ONLY	GSE13917	GPL570
GSM381213	RA_TREAT	GSE15258	GPL570	GSM389703	RA_ONLY	GSE15573	GPL6102
GSM381214	RA_TREAT	GSE15258	GPL570	GSM389704	RA_ONLY	GSE15573	GPL6102
GSM381215	RA_TREAT	GSE15258	GPL570	GSM389706	RA_ONLY	GSE15573	GPL6102
GSM381216	RA_TREAT	GSE15258	GPL570	GSM389708	RA_ONLY	GSE15573	GPL6102
GSM381217	RA_TREAT	GSE15258	GPL570	GSM389711	RA_ONLY	GSE15573	GPL6102
GSM381218	RA_TREAT	GSE15258	GPL570	GSM389714	RA_ONLY	GSE15573	GPL6102
GSM381219	RA_TREAT	GSE15258	GPL570	GSM389716	RA_ONLY	GSE15573	GPL6102
GSM381220	RA_TREAT	GSE15258	GPL570	GSM389717	RA_ONLY	GSE15573	GPL6102
GSM381221	RA_TREAT	GSE15258	GPL570	GSM389719	RA_ONLY	GSE15573	GPL6102
GSM381222	RA_TREAT	GSE15258	GPL570	GSM389721	RA_ONLY	GSE15573	GPL6102
GSM381223	RA_TREAT	GSE15258	GPL570	GSM389722	RA_ONLY	GSE15573	GPL6102
GSM381224	RA_TREAT	GSE15258	GPL570	GSM389724	RA_ONLY	GSE15573	GPL6102
GSM381225	RA_TREAT	GSE15258	GPL570	GSM389726	RA_ONLY	GSE15573	GPL6102
GSM381226	RA_TREAT	GSE15258	GPL570	GSM389727	RA_ONLY	GSE15573	GPL6102
GSM381227	RA_TREAT	GSE15258	GPL570	GSM389730	RA_ONLY	GSE15573	GPL6102
GSM381228	RA_TREAT	GSE15258	GPL570	GSM389731	RA_ONLY	GSE15573	GPL6102
GSM381229	RA_TREAT	GSE15258	GPL570	GSM389733	RA_ONLY	GSE15573	GPL6102
GSM381230	RA_TREAT	GSE15258	GPL570	GSM389735	RA_ONLY	GSE15573	GPL6102
GSM381231	RA_TREAT	GSE15258	GPL570	GSM390940	RA_ONLY	GSE15615	GPL570
GSM381232	RA_TREAT	GSE15258	GPL570	GSM546092	RA_ONLY	GSE21959	GPL4133
GSM381233	RA_TREAT	GSE15258	GPL570	GSM546093	RA_ONLY	GSE21959	GPL4133
GSM381234	RA_TREAT	GSE15258	GPL570	GSM546094	RA_ONLY	GSE21959	GPL4133
GSM381235	RA_TREAT	GSE15258	GPL570	GSM546095	RA_ONLY	GSE21959	GPL4133
GSM381236	RA_TREAT	GSE15258	GPL570	GSM546096	RA_ONLY	GSE21959	GPL4133
GSM381237	RA_TREAT	GSE15258	GPL570	GSM546097	RA_ONLY	GSE21959	GPL4133
GSM381238	RA_TREAT	GSE15258	GPL570	GSM546106	RA_ONLY	GSE21959	GPL4133
GSM381239	RA_TREAT	GSE15258	GPL570	GSM546107	RA_ONLY	GSE21959	GPL4133
GSM381240	RA_TREAT	GSE15258	GPL570	GSM546108	RA_ONLY	GSE21959	GPL4133
GSM381241	RA_TREAT	GSE15258	GPL570	GSM546109	RA_ONLY	GSE21959	GPL4133
GSM381242	RA_TREAT	GSE15258	GPL570	GSM546110	RA_ONLY	GSE21959	GPL4133
GSM381243	RA_TREAT	GSE15258	GPL570	GSM546111	RA_ONLY	GSE21959	GPL4133
GSM381244	RA_TREAT	GSE15258	GPL570	GSM546112	RA_ONLY	GSE21959	GPL4133
GSM381245	RA_TREAT	GSE15258	GPL570	GSM546113	RA_ONLY	GSE21959	GPL4133
GSM381246	RA_TREAT	GSE15258	GPL570	GSM546114	RA_ONLY	GSE21959	GPL4133
GSM381247	RA_TREAT	GSE15258	GPL570	GSM546115	RA_ONLY	GSE21959	GPL4133
GSM381248	RA_TREAT	GSE15258	GPL570	GSM546116	RA_ONLY	GSE21959	GPL4133
GSM381249	RA_TREAT	GSE15258	GPL570	GSM546117	RA_ONLY	GSE21959	GPL4133

GSM381250	RA_TREAT	GSE15258	GPL570	GSM566728	RA_ONLY	GSE22956	GPL6947
GSM381251	RA_TREAT	GSE15258	GPL570	GSM566731	RA_ONLY	GSE22956	GPL6947
GSM381252	RA_TREAT	GSE15258	GPL570	GSM566734	RA_ONLY	GSE22956	GPL6947
GSM381253	RA_TREAT	GSE15258	GPL570	GSM566737	RA_ONLY	GSE22956	GPL6947
GSM381254	RA_TREAT	GSE15258	GPL570	GSM607508	RA_ONLY	GSE24742	GPL570
GSM381255	RA_TREAT	GSE15258	GPL570	GSM609031	RA_ONLY	GSE24742	GPL570
GSM381256	RA_TREAT	GSE15258	GPL570	GSM609033	RA_ONLY	GSE24742	GPL570
GSM381257	RA_TREAT	GSE15258	GPL570	GSM609035	RA_ONLY	GSE24742	GPL570
GSM381258	RA_TREAT	GSE15258	GPL570	GSM609037	RA_ONLY	GSE24742	GPL570
GSM381259	RA_TREAT	GSE15258	GPL570	GSM609386	RA_ONLY	GSE24742	GPL570
GSM381260	RA_TREAT	GSE15258	GPL570	GSM609388	RA_ONLY	GSE24742	GPL570
GSM381261	RA_TREAT	GSE15258	GPL570	GSM609390	RA_ONLY	GSE24742	GPL570
GSM381262	RA_TREAT	GSE15258	GPL570	GSM609392	RA_ONLY	GSE24742	GPL570
GSM381263	RA_TREAT	GSE15258	GPL570	GSM609394	RA_ONLY	GSE24742	GPL570
GSM381264	RA_TREAT	GSE15258	GPL570	GSM609396	RA_ONLY	GSE24742	GPL570
GSM381265	RA_TREAT	GSE15258	GPL570	GSM609398	RA_ONLY	GSE24742	GPL570
GSM381266	RA_TREAT	GSE15258	GPL570	GSM618155	RA_ONLY	GSE25160	GPL6244
GSM381267	RA_TREAT	GSE15258	GPL570	GSM618156	RA_ONLY	GSE25160	GPL6244
GSM381268	RA_TREAT	GSE15258	GPL570	GSM618157	RA_ONLY	GSE25160	GPL6244
GSM381269	RA_TREAT	GSE15258	GPL570	GSM618158	RA_ONLY	GSE25160	GPL6244
GSM381270	RA_TREAT	GSE15258	GPL570	GSM618159	RA_ONLY	GSE25160	GPL6244
GSM381271	RA_TREAT	GSE15258	GPL570	GSM618160	RA_ONLY	GSE25160	GPL6244
GSM381272	RA_TREAT	GSE15258	GPL570	GSM618161	RA_ONLY	GSE25160	GPL6244
GSM381273	RA_TREAT	GSE15258	GPL570	GSM618162	RA_ONLY	GSE25160	GPL6244
GSM381274	RA_TREAT	GSE15258	GPL570	GSM618163	RA_ONLY	GSE25160	GPL6244
GSM381275	RA_TREAT	GSE15258	GPL570	GSM618164	RA_ONLY	GSE25160	GPL6244
GSM381276	RA_TREAT	GSE15258	GPL570	GSM618165	RA_ONLY	GSE25160	GPL6244
GSM381277	RA_TREAT	GSE15258	GPL570	GSM618166	RA_ONLY	GSE25160	GPL6244
GSM381278	RA_TREAT	GSE15258	GPL570	GSM618167	RA_ONLY	GSE25160	GPL6244
GSM381279	RA_TREAT	GSE15258	GPL570	GSM677339	RA_ONLY	GSE27390	GPL570
GSM390153	RA_TREAT	GSE15602	GPL570	GSM677340	RA_ONLY	GSE27390	GPL570
GSM390154	RA_TREAT	GSE15602	GPL570	GSM677341	RA_ONLY	GSE27390	GPL570
GSM390155	RA_TREAT	GSE15602	GPL570	GSM677342	RA_ONLY	GSE27390	GPL570
GSM390156	RA_TREAT	GSE15602	GPL570	GSM677343	RA_ONLY	GSE27390	GPL570
GSM390157	RA_TREAT	GSE15602	GPL570	GSM677344	RA_ONLY	GSE27390	GPL570
GSM390158	RA_TREAT	GSE15602	GPL570	GSM677345	RA_ONLY	GSE27390	GPL570
GSM390159	RA_TREAT	GSE15602	GPL570	GSM677346	RA_ONLY	GSE27390	GPL570
GSM390160	RA_TREAT	GSE15602	GPL570	GSM677347	RA_ONLY	GSE27390	GPL570
GSM390170	RA_TREAT	GSE15602	GPL570	GSM737461	RA_ONLY	GSE29746	GPL4133
GSM390171	RA_TREAT	GSE15602	GPL570	GSM737462	RA_ONLY	GSE29746	GPL4133
GSM390173	RA_TREAT	GSE15602	GPL570	GSM737463	RA_ONLY	GSE29746	GPL4133
GSM390843	RA_TREAT	GSE15615	GPL570	GSM737464	RA_ONLY	GSE29746	GPL4133
GSM390941	RA_TREAT	GSE15615	GPL570	GSM737465	RA_ONLY	GSE29746	GPL4133
GSM390942	RA_TREAT	GSE15615	GPL570	GSM737466	RA_ONLY	GSE29746	GPL4133
GSM390943	RA_TREAT	GSE15615	GPL570	GSM737467	RA_ONLY	GSE29746	GPL4133
GSM390944	RA_TREAT	GSE15615	GPL570	GSM737468	RA_ONLY	GSE29746	GPL4133
GSM34398	RA_TREAT	GSE1919	GPL91	GSM737469	RA_ONLY	GSE29746	GPL4133

GSM34399	RA_TREAT	GSE1919	GPL91	GSM743016	RA_ONLY	GSE30023	GPL1708
GSM34400	RA_TREAT	GSE1919	GPL91	GSM743018	RA_ONLY	GSE30023	GPL1708
GSM34401	RA_TREAT	GSE1919	GPL91	GSM743019	RA_ONLY	GSE30023	GPL1708
GSM34402	RA_TREAT	GSE1919	GPL91	GSM899014	RA_ONLY	GSE36700	GPL570
GSM519451	RA_TREAT	GSE20690	GPL4133	GSM899015	RA_ONLY	GSE36700	GPL570
GSM519452	RA_TREAT	GSE20690	GPL4133	GSM899016	RA_ONLY	GSE36700	GPL570
GSM519453	RA_TREAT	GSE20690	GPL4133	GSM899017	RA_ONLY	GSE36700	GPL570
GSM519454	RA_TREAT	GSE20690	GPL4133	GSM899018	RA_ONLY	GSE36700	GPL570
GSM519455	RA_TREAT	GSE20690	GPL4133	GSM899019	RA_ONLY	GSE36700	GPL570
GSM519456	RA_TREAT	GSE20690	GPL4133	GSM899020	RA_ONLY	GSE36700	GPL570
GSM519457	RA_TREAT	GSE20690	GPL4133	GSM1037336	RA_ONLY	GSE42296	GPL6244
GSM519458	RA_TREAT	GSE20690	GPL4133	GSM1037337	RA_ONLY	GSE42296	GPL6244
GSM519459	RA_TREAT	GSE20690	GPL4133	GSM1037338	RA_ONLY	GSE42296	GPL6244
GSM519460	RA_TREAT	GSE20690	GPL4133	GSM1037339	RA_ONLY	GSE42296	GPL6244
GSM519461	RA_TREAT	GSE20690	GPL4133	GSM1037340	RA_ONLY	GSE42296	GPL6244
GSM519462	RA_TREAT	GSE20690	GPL4133	GSM1037341	RA_ONLY	GSE42296	GPL6244
GSM519463	RA_TREAT	GSE20690	GPL4133	GSM1037342	RA_ONLY	GSE42296	GPL6244
GSM519464	RA_TREAT	GSE20690	GPL4133	GSM1037343	RA_ONLY	GSE42296	GPL6244
GSM519465	RA_TREAT	GSE20690	GPL4133	GSM1037344	RA_ONLY	GSE42296	GPL6244
GSM519466	RA_TREAT	GSE20690	GPL4133	GSM1037345	RA_ONLY	GSE42296	GPL6244
GSM519467	RA_TREAT	GSE20690	GPL4133	GSM1037346	RA_ONLY	GSE42296	GPL6244
GSM519468	RA_TREAT	GSE20690	GPL4133	GSM1037347	RA_ONLY	GSE42296	GPL6244
GSM519469	RA_TREAT	GSE20690	GPL4133	GSM1037348	RA_ONLY	GSE42296	GPL6244
GSM519470	RA_TREAT	GSE20690	GPL4133	GSM1037349	RA_ONLY	GSE42296	GPL6244
GSM519471	RA_TREAT	GSE20690	GPL4133	GSM1037350	RA_ONLY	GSE42296	GPL6244
GSM519472	RA_TREAT	GSE20690	GPL4133	GSM1037351	RA_ONLY	GSE42296	GPL6244
GSM519473	RA_TREAT	GSE20690	GPL4133	GSM1037352	RA_ONLY	GSE42296	GPL6244
GSM519474	RA_TREAT	GSE20690	GPL4133	GSM1037353	RA_ONLY	GSE42296	GPL6244
GSM519475	RA_TREAT	GSE20690	GPL4133	GSM1037354	RA_ONLY	GSE42296	GPL6244
GSM519476	RA_TREAT	GSE20690	GPL4133	GSM1184435	RA_ONLY	GSE48780	GPL570
GSM519477	RA_TREAT	GSE20690	GPL4133	GSM1184436	RA_ONLY	GSE48780	GPL570
GSM519478	RA_TREAT	GSE20690	GPL4133	GSM1184437	RA_ONLY	GSE48780	GPL570
GSM519479	RA_TREAT	GSE20690	GPL4133	GSM1184438	RA_ONLY	GSE48780	GPL570
GSM519480	RA_TREAT	GSE20690	GPL4133	GSM1184439	RA_ONLY	GSE48780	GPL570
GSM519481	RA_TREAT	GSE20690	GPL4133	GSM1184440	RA_ONLY	GSE48780	GPL570
GSM519482	RA_TREAT	GSE20690	GPL4133	GSM1184441	RA_ONLY	GSE48780	GPL570
GSM519483	RA_TREAT	GSE20690	GPL4133	GSM1184442	RA_ONLY	GSE48780	GPL570
GSM519484	RA_TREAT	GSE20690	GPL4133	GSM1184443	RA_ONLY	GSE48780	GPL570
GSM519485	RA_TREAT	GSE20690	GPL4133	GSM1184444	RA_ONLY	GSE48780	GPL570
GSM519486	RA_TREAT	GSE20690	GPL4133	GSM1184445	RA_ONLY	GSE48780	GPL570
GSM519487	RA_TREAT	GSE20690	GPL4133	GSM1184446	RA_ONLY	GSE48780	GPL570
GSM519488	RA_TREAT	GSE20690	GPL4133	GSM1184447	RA_ONLY	GSE48780	GPL570
GSM519489	RA_TREAT	GSE20690	GPL4133	GSM1184448	RA_ONLY	GSE48780	GPL570
GSM519490	RA_TREAT	GSE20690	GPL4133	GSM1184449	RA_ONLY	GSE48780	GPL570
GSM519491	RA_TREAT	GSE20690	GPL4133	GSM1184450	RA_ONLY	GSE48780	GPL570
GSM519492	RA_TREAT	GSE20690	GPL4133	GSM1184451	RA_ONLY	GSE48780	GPL570
GSM519493	RA_TREAT	GSE20690	GPL4133	GSM1184452	RA_ONLY	GSE48780	GPL570

GSM519494	RA_TREAT	GSE20690	GPL4133	GSM1184453	RA_ONLY	GSE48780	GPL570
GSM519495	RA_TREAT	GSE20690	GPL4133	GSM1184454	RA_ONLY	GSE48780	GPL570
GSM519496	RA_TREAT	GSE20690	GPL4133	GSM1184455	RA_ONLY	GSE48780	GPL570
GSM519497	RA_TREAT	GSE20690	GPL4133	GSM1184456	RA_ONLY	GSE48780	GPL570
GSM519498	RA_TREAT	GSE20690	GPL4133	GSM1184457	RA_ONLY	GSE48780	GPL570
GSM519499	RA_TREAT	GSE20690	GPL4133	GSM1184458	RA_ONLY	GSE48780	GPL570
GSM519500	RA_TREAT	GSE20690	GPL4133	GSM1184459	RA_ONLY	GSE48780	GPL570
GSM519501	RA_TREAT	GSE20690	GPL4133	GSM1184460	RA_ONLY	GSE48780	GPL570
GSM519502	RA_TREAT	GSE20690	GPL4133	GSM1184461	RA_ONLY	GSE48780	GPL570
GSM519503	RA_TREAT	GSE20690	GPL4133	GSM1184462	RA_ONLY	GSE48780	GPL570
GSM519504	RA_TREAT	GSE20690	GPL4133	GSM1184463	RA_ONLY	GSE48780	GPL570
GSM519505	RA_TREAT	GSE20690	GPL4133	GSM1184464	RA_ONLY	GSE48780	GPL570
GSM519506	RA_TREAT	GSE20690	GPL4133	GSM1184465	RA_ONLY	GSE48780	GPL570
GSM519507	RA_TREAT	GSE20690	GPL4133	GSM1184466	RA_ONLY	GSE48780	GPL570
GSM519508	RA_TREAT	GSE20690	GPL4133	GSM1184467	RA_ONLY	GSE48780	GPL570
GSM519509	RA_TREAT	GSE20690	GPL4133	GSM1184468	RA_ONLY	GSE48780	GPL570
GSM519510	RA_TREAT	GSE20690	GPL4133	GSM1184469	RA_ONLY	GSE48780	GPL570
GSM519511	RA_TREAT	GSE20690	GPL4133	GSM1184470	RA_ONLY	GSE48780	GPL570
GSM519512	RA_TREAT	GSE20690	GPL4133	GSM1184471	RA_ONLY	GSE48780	GPL570
GSM519513	RA_TREAT	GSE20690	GPL4133	GSM1184472	RA_ONLY	GSE48780	GPL570
GSM519514	RA_TREAT	GSE20690	GPL4133	GSM1184473	RA_ONLY	GSE48780	GPL570
GSM519515	RA_TREAT	GSE20690	GPL4133	GSM1184474	RA_ONLY	GSE48780	GPL570
GSM519516	RA_TREAT	GSE20690	GPL4133	GSM1184475	RA_ONLY	GSE48780	GPL570
GSM519517	RA_TREAT	GSE20690	GPL4133	GSM1184476	RA_ONLY	GSE48780	GPL570
GSM519518	RA_TREAT	GSE20690	GPL4133	GSM1184477	RA_ONLY	GSE48780	GPL570
GSM607509	RA_TREAT	GSE24742	GPL570	GSM1184478	RA_ONLY	GSE48780	GPL570
GSM609032	RA_TREAT	GSE24742	GPL570	GSM1184479	RA_ONLY	GSE48780	GPL570
GSM609034	RA_TREAT	GSE24742	GPL570	GSM1184480	RA_ONLY	GSE48780	GPL570
GSM609036	RA_TREAT	GSE24742	GPL570	GSM1184481	RA_ONLY	GSE48780	GPL570
GSM609038	RA_TREAT	GSE24742	GPL570	GSM1184482	RA_ONLY	GSE48780	GPL570
GSM609387	RA_TREAT	GSE24742	GPL570	GSM1184483	RA_ONLY	GSE48780	GPL570
GSM609389	RA_TREAT	GSE24742	GPL570	GSM1184484	RA_ONLY	GSE48780	GPL570
GSM609391	RA_TREAT	GSE24742	GPL570	GSM1184485	RA_ONLY	GSE48780	GPL570
GSM609393	RA_TREAT	GSE24742	GPL570	GSM1184486	RA_ONLY	GSE48780	GPL570
GSM609395	RA_TREAT	GSE24742	GPL570	GSM1184487	RA_ONLY	GSE48780	GPL570
GSM609397	RA_TREAT	GSE24742	GPL570	GSM1184488	RA_ONLY	GSE48780	GPL570
GSM609399	RA_TREAT	GSE24742	GPL570	GSM1184489	RA_ONLY	GSE48780	GPL570
GSM618168	RA_TREAT	GSE25160	GPL6244	GSM1184490	RA_ONLY	GSE48780	GPL570
GSM618169	RA_TREAT	GSE25160	GPL6244	GSM1184491	RA_ONLY	GSE48780	GPL570
GSM618170	RA_TREAT	GSE25160	GPL6244	GSM1184492	RA_ONLY	GSE48780	GPL570
GSM618171	RA_TREAT	GSE25160	GPL6244	GSM1184493	RA_ONLY	GSE48780	GPL570
GSM618172	RA_TREAT	GSE25160	GPL6244	GSM1184494	RA_ONLY	GSE48780	GPL570
GSM618173	RA_TREAT	GSE25160	GPL6244	GSM1184495	RA_ONLY	GSE48780	GPL570
GSM618174	RA_TREAT	GSE25160	GPL6244	GSM1184496	RA_ONLY	GSE48780	GPL570
GSM618175	RA_TREAT	GSE25160	GPL6244	GSM1184497	RA_ONLY	GSE48780	GPL570
GSM618176	RA_TREAT	GSE25160	GPL6244	GSM1184498	RA_ONLY	GSE48780	GPL570
GSM618177	RA_TREAT	GSE25160	GPL6244	GSM1184499	RA_ONLY	GSE48780	GPL570

GSM618178	RA_TREAT	GSE25160	GPL6244	GSM1184500	RA_ONLY	GSE48780	GPL570
GSM618179	RA_TREAT	GSE25160	GPL6244	GSM1184501	RA_ONLY	GSE48780	GPL570
GSM618180	RA_TREAT	GSE25160	GPL6244	GSM1184502	RA_ONLY	GSE48780	GPL570
GSM1037355	RA_TREAT	GSE42296	GPL6244	GSM1184503	RA_ONLY	GSE48780	GPL570
GSM1037356	RA_TREAT	GSE42296	GPL6244	GSM1184504	RA_ONLY	GSE48780	GPL570
GSM1037357	RA_TREAT	GSE42296	GPL6244	GSM1184505	RA_ONLY	GSE48780	GPL570
GSM1037358	RA_TREAT	GSE42296	GPL6244	GSM1184506	RA_ONLY	GSE48780	GPL570
GSM1037359	RA_TREAT	GSE42296	GPL6244	GSM1184507	RA_ONLY	GSE48780	GPL570
GSM1037360	RA_TREAT	GSE42296	GPL6244	GSM1184508	RA_ONLY	GSE48780	GPL570
GSM1037361	RA_TREAT	GSE42296	GPL6244	GSM1184509	RA_ONLY	GSE48780	GPL570
GSM1037362	RA_TREAT	GSE42296	GPL6244	GSM1184510	RA_ONLY	GSE48780	GPL570
GSM1037363	RA_TREAT	GSE42296	GPL6244	GSM1184511	RA_ONLY	GSE48780	GPL570
GSM1037364	RA_TREAT	GSE42296	GPL6244	GSM1184512	RA_ONLY	GSE48780	GPL570
GSM1037365	RA_TREAT	GSE42296	GPL6244	GSM1184513	RA_ONLY	GSE48780	GPL570
GSM1037366	RA_TREAT	GSE42296	GPL6244	GSM1184514	RA_ONLY	GSE48780	GPL570
GSM1037367	RA_TREAT	GSE42296	GPL6244	GSM1184515	RA_ONLY	GSE48780	GPL570
GSM1037368	RA_TREAT	GSE42296	GPL6244	GSM1184516	RA_ONLY	GSE48780	GPL570
GSM1037369	RA_TREAT	GSE42296	GPL6244	GSM1184517	RA_ONLY	GSE48780	GPL570
GSM1037370	RA_TREAT	GSE42296	GPL6244	GSM1202493	RA_ONLY	GSE49604	GPL8432
GSM1037371	RA_TREAT	GSE42296	GPL6244	GSM1202494	RA_ONLY	GSE49604	GPL8432
GSM1037372	RA_TREAT	GSE42296	GPL6244	GSM1202495	RA_ONLY	GSE49604	GPL8432
GSM1037373	RA_TREAT	GSE42296	GPL6244	GSM1202496	RA_ONLY	GSE49604	GPL8432
GSM1320152	RA_TREAT	GSE54629	GPL6244	GSM1202497	RA_ONLY	GSE49604	GPL8432
GSM1320153	RA_TREAT	GSE54629	GPL6244	GSM1202498	RA_ONLY	GSE49604	GPL8432
GSM1320154	RA_TREAT	GSE54629	GPL6244	GSM1360956	RA_ONLY	GSE56409	GPL570
GSM1320155	RA_TREAT	GSE54629	GPL6244	GSM1360957	RA_ONLY	GSE56409	GPL570
GSM1320156	RA_TREAT	GSE54629	GPL6244	GSM1360958	RA_ONLY	GSE56409	GPL570
GSM1320157	RA_TREAT	GSE54629	GPL6244	GSM1360959	RA_ONLY	GSE56409	GPL570
GSM1320158	RA_TREAT	GSE54629	GPL6244	GSM1360960	RA_ONLY	GSE56409	GPL570
GSM1320159	RA_TREAT	GSE54629	GPL6244	GSM1360961	RA_ONLY	GSE56409	GPL570
GSM1320160	RA_TREAT	GSE54629	GPL6244	GSM1360962	RA_ONLY	GSE56409	GPL570
GSM1320161	RA_TREAT	GSE54629	GPL6244	GSM1360963	RA_ONLY	GSE56409	GPL570
GSM1320162	RA_TREAT	GSE54629	GPL6244	GSM1360964	RA_ONLY	GSE56409	GPL570
GSM1320163	RA_TREAT	GSE54629	GPL6244	GSM1360965	RA_ONLY	GSE56409	GPL570
GSM1320164	RA_TREAT	GSE54629	GPL6244	GSM1360966	RA_ONLY	GSE56409	GPL570
GSM1320165	RA_TREAT	GSE54629	GPL6244	GSM1360967	RA_ONLY	GSE56409	GPL570
GSM1320166	RA_TREAT	GSE54629	GPL6244	GSM1360968	RA_ONLY	GSE56409	GPL570
GSM1320167	RA_TREAT	GSE54629	GPL6244	GSM1360969	RA_ONLY	GSE56409	GPL570
GSM1320168	RA_TREAT	GSE54629	GPL6244	GSM1360970	RA_ONLY	GSE56409	GPL570
GSM1320169	RA_TREAT	GSE54629	GPL6244	GSM1360971	RA_ONLY	GSE56409	GPL570
GSM1320170	RA_TREAT	GSE54629	GPL6244	GSM1360972	RA_ONLY	GSE56409	GPL570
GSM1320171	RA_TREAT	GSE54629	GPL6244	GSM1360973	RA_ONLY	GSE56409	GPL570
GSM1320172	RA_TREAT	GSE54629	GPL6244	GSM1360974	RA_ONLY	GSE56409	GPL570
GSM1320173	RA_TREAT	GSE54629	GPL6244	GSM1360975	RA_ONLY	GSE56409	GPL570
GSM1320174	RA_TREAT	GSE54629	GPL6244	GSM1360976	RA_ONLY	GSE56409	GPL570
GSM1320175	RA_TREAT	GSE54629	GPL6244	GSM1360977	RA_ONLY	GSE56409	GPL570
GSM1320176	RA_TREAT	GSE54629	GPL6244	GSM1360988	RA_ONLY	GSE56409	GPL570

GSM1320177	RA_TREAT	GSE54629	GPL6244	GSM1360989	RA_ONLY	GSE56409	GPL570
GSM1320178	RA_TREAT	GSE54629	GPL6244	GSM1360990	RA_ONLY	GSE56409	GPL570
GSM1320179	RA_TREAT	GSE54629	GPL6244	GSM1360991	RA_ONLY	GSE56409	GPL570
GSM1320180	RA_TREAT	GSE54629	GPL6244	GSM1360992	RA_ONLY	GSE56409	GPL570
GSM1320181	RA_TREAT	GSE54629	GPL6244	GSM1360993	RA_ONLY	GSE56409	GPL570
GSM1320182	RA_TREAT	GSE54629	GPL6244	GSM1360994	RA_ONLY	GSE56409	GPL570
GSM1320183	RA_TREAT	GSE54629	GPL6244	GSM1360995	RA_ONLY	GSE56409	GPL570
GSM1320184	RA_TREAT	GSE54629	GPL6244	GSM1360996	RA_ONLY	GSE56409	GPL570
GSM1320185	RA_TREAT	GSE54629	GPL6244	GSM1360997	RA_ONLY	GSE56409	GPL570
GSM1320186	RA_TREAT	GSE54629	GPL6244	GSM1360998	RA_ONLY	GSE56409	GPL570
GSM1320187	RA_TREAT	GSE54629	GPL6244	GSM1360999	RA_ONLY	GSE56409	GPL570
GSM1320188	RA_TREAT	GSE54629	GPL6244	GSM1361000	RA_ONLY	GSE56409	GPL570
GSM1320189	RA_TREAT	GSE54629	GPL6244	GSM1361001	RA_ONLY	GSE56409	GPL570
GSM1320190	RA_TREAT	GSE54629	GPL6244	GSM1361002	RA_ONLY	GSE56409	GPL570
GSM1320191	RA_TREAT	GSE54629	GPL6244	GSM1361003	RA_ONLY	GSE56409	GPL570
GSM1320192	RA_TREAT	GSE54629	GPL6244	GSM1361004	RA_ONLY	GSE56409	GPL570
GSM1320193	RA_TREAT	GSE54629	GPL6244	GSM1361005	RA_ONLY	GSE56409	GPL570
GSM1320194	RA_TREAT	GSE54629	GPL6244	GSM1361006	RA_ONLY	GSE56409	GPL570
GSM1320195	RA_TREAT	GSE54629	GPL6244	GSM1361007	RA_ONLY	GSE56409	GPL570
GSM1320196	RA_TREAT	GSE54629	GPL6244	GSM1361008	RA_ONLY	GSE56409	GPL570
GSM1320197	RA_TREAT	GSE54629	GPL6244	GSM1361009	RA_ONLY	GSE56409	GPL570
GSM1320198	RA_TREAT	GSE54629	GPL6244	GSM1361010	RA_ONLY	GSE56409	GPL570
GSM1320199	RA_TREAT	GSE54629	GPL6244	GSM1361011	RA_ONLY	GSE56409	GPL570
GSM1320200	RA_TREAT	GSE54629	GPL6244	GSM1361024	RA_ONLY	GSE56409	GPL570
GSM1320201	RA_TREAT	GSE54629	GPL6244	GSM1361025	RA_ONLY	GSE56409	GPL570
GSM1320202	RA_TREAT	GSE54629	GPL6244	GSM1361026	RA_ONLY	GSE56409	GPL570
GSM1320203	RA_TREAT	GSE54629	GPL6244	GSM1361027	RA_ONLY	GSE56409	GPL570
GSM1320204	RA_TREAT	GSE54629	GPL6244	GSM1361028	RA_ONLY	GSE56409	GPL570
GSM1320205	RA_TREAT	GSE54629	GPL6244	GSM1361029	RA_ONLY	GSE56409	GPL570
GSM1320206	RA_TREAT	GSE54629	GPL6244	GSM1361030	RA_ONLY	GSE56409	GPL570
GSM1320207	RA_TREAT	GSE54629	GPL6244	GSM1361031	RA_ONLY	GSE56409	GPL570
GSM1320208	RA_TREAT	GSE54629	GPL6244	GSM1361032	RA_ONLY	GSE56409	GPL570
GSM1320209	RA_TREAT	GSE54629	GPL6244	GSM1361033	RA_ONLY	GSE56409	GPL570
GSM1320210	RA_TREAT	GSE54629	GPL6244	GSM1361034	RA_ONLY	GSE56409	GPL570
GSM1320211	RA_TREAT	GSE54629	GPL6244	GSM1361035	RA_ONLY	GSE56409	GPL570
GSM1320212	RA_TREAT	GSE54629	GPL6244	GSM1361036	RA_ONLY	GSE56409	GPL570
GSM1320213	RA_TREAT	GSE54629	GPL6244	GSM1361037	RA_ONLY	GSE56409	GPL570
GSM1320214	RA_TREAT	GSE54629	GPL6244	GSM1361038	RA_ONLY	GSE56409	GPL570
GSM1320215	RA_TREAT	GSE54629	GPL6244	GSM1361039	RA_ONLY	GSE56409	GPL570
GSM1320216	RA_TREAT	GSE54629	GPL6244	GSM1361040	RA_ONLY	GSE56409	GPL570
GSM1320217	RA_TREAT	GSE54629	GPL6244	GSM1361041	RA_ONLY	GSE56409	GPL570
GSM1320218	RA_TREAT	GSE54629	GPL6244	GSM1361042	RA_ONLY	GSE56409	GPL570
GSM1320219	RA_TREAT	GSE54629	GPL6244	GSM1361043	RA_ONLY	GSE56409	GPL570
GSM1320220	RA_TREAT	GSE54629	GPL6244	GSM1361044	RA_ONLY	GSE56409	GPL570
GSM1320221	RA_TREAT	GSE54629	GPL6244	GSM1361045	RA_ONLY	GSE56409	GPL570
GSM1320222	RA_TREAT	GSE54629	GPL6244	GSM1381406	RA_ONLY	GSE57386	GPL13158
GSM1320223	RA_TREAT	GSE54629	GPL6244	GSM1381407	RA_ONLY	GSE57386	GPL13158

GSM1320224	RA_TREAT	GSE54629	GPL6244	GSM1381408	RA_ONLY	GSE57386	GPL13158
GSM1320225	RA_TREAT	GSE54629	GPL6244	GSM1381609	RA_ONLY	GSE57383	GPL13158
GSM1320226	RA_TREAT	GSE54629	GPL6244	GSM1381616	RA_ONLY	GSE57383	GPL13158
GSM1320227	RA_TREAT	GSE54629	GPL6244	GSM1381620	RA_ONLY	GSE57383	GPL13158
GSM1320228	RA_TREAT	GSE54629	GPL6244	GSM1381621	RA_ONLY	GSE57383	GPL13158
GSM1320229	RA_TREAT	GSE54629	GPL6244	GSM1381624	RA_ONLY	GSE57383	GPL13158
GSM1320230	RA_TREAT	GSE54629	GPL6244	GSM1381629	RA_ONLY	GSE57383	GPL13158
GSM1320231	RA_TREAT	GSE54629	GPL6244	GSM1381632	RA_ONLY	GSE57383	GPL13158
GSM1320232	RA_TREAT	GSE54629	GPL6244	GSM1381634	RA_ONLY	GSE57383	GPL13158
GSM1320233	RA_TREAT	GSE54629	GPL6244	GSM1381635	RA_ONLY	GSE57383	GPL13158
GSM1320234	RA_TREAT	GSE54629	GPL6244	GSM1382124	RA_ONLY	GSE57405	GPL13158
GSM1320235	RA_TREAT	GSE54629	GPL6244	GSM1382125	RA_ONLY	GSE57405	GPL13158
GSM1320236	RA_TREAT	GSE54629	GPL6244	GSM1382126	RA_ONLY	GSE57405	GPL13158
GSM1320237	RA_TREAT	GSE54629	GPL6244	GSM1382127	RA_ONLY	GSE57405	GPL13158
GSM1320238	RA_TREAT	GSE54629	GPL6244	GSM1382128	RA_ONLY	GSE57405	GPL13158
GSM1320239	RA_TREAT	GSE54629	GPL6244	GSM1382129	RA_ONLY	GSE57405	GPL13158
GSM1320240	RA_TREAT	GSE54629	GPL6244	GSM1382130	RA_ONLY	GSE57405	GPL13158
GSM1320241	RA_TREAT	GSE54629	GPL6244	GSM1382131	RA_ONLY	GSE57405	GPL13158
GSM1320242	RA_TREAT	GSE54629	GPL6244	GSM1382132	RA_ONLY	GSE57405	GPL13158
GSM1320243	RA_TREAT	GSE54629	GPL6244	GSM1562202	RA_ONLY	GSE63995	GPL570
GSM1320244	RA_TREAT	GSE54629	GPL6244	GSM1562204	RA_ONLY	GSE63995	GPL570
GSM1320245	RA_TREAT	GSE54629	GPL6244	GSM1562206	RA_ONLY	GSE63995	GPL570
GSM1320246	RA_TREAT	GSE54629	GPL6244	GSM1562208	RA_ONLY	GSE63995	GPL570
GSM1320247	RA_TREAT	GSE54629	GPL6244	GSM119291	RA_ONLY	GSE7524	GPL96
GSM1320248	RA_TREAT	GSE54629	GPL6244	GSM119292	RA_ONLY	GSE7524	GPL96
GSM1320249	RA_TREAT	GSE54629	GPL6244	GSM183695	RA_ONLY	GSE7669	GPL8300
